# Global epidemiology, genetic environment, risk factors and therapeutic prospects of *mcr* genes: A current and emerging update

**DOI:** 10.3389/fcimb.2022.941358

**Published:** 2022-08-26

**Authors:** Masego Mmatli, Nontombi Marylucy Mbelle, John Osei Sekyere

**Affiliations:** ^1^ Department of Medical Microbiology, School of Medicine, University of Pretoria, Pretoria, South Africa; ^2^ Department of Microbiology and Immunology, Indiana University School of Medicine-Northwest, Gary, IN, United States; ^3^ Department of Dermatology, School of Medicine, University of Pretoria, Pretoria, South Africa

**Keywords:** mcr-1, pandrug resistance, colistin resistance, polymyxins, crystal structure, MCR activity, risk factors, *Enterobacteriaceae*

## Abstract

**Background:**

Mobile colistin resistance (*mcr*) genes modify Lipid A molecules of the lipopolysaccharide, changing the overall charge of the outer membrane.

**Results and discussion:**

Ten *mcr* genes have been described to date within eleven Enterobacteriaceae species, with *Escherichia coli*, *Klebsiella pneumoniae*, and *Salmonella* species being the most predominant. They are present worldwide in 72 countries, with animal specimens currently having the highest incidence, due to the use of colistin in poultry for promoting growth and treating intestinal infections. The wide dissemination of *mcr* from food animals to meat, manure, the environment, and wastewater samples has increased the risk of transmission to humans *via* foodborne and vector-borne routes. The stability and spread of *mcr* genes were mediated by mobile genetic elements such as the IncHI_2_ conjugative plasmid, which is associated with multiple *mcr* genes and other antibiotic resistance genes. The cost of acquiring *mcr* is reduced by compensatory adaptation mechanisms. MCR proteins are well conserved structurally and *via* enzymatic action. Thus, therapeutics found effective against MCR-1 should be tested against the remaining MCR proteins.

**Conclusion:**

The dissemination of *mcr* genes into the clinical setting, is threatening public health by limiting therapeutics options available. Combination therapies are a promising option for managing and treating colistin-resistant *Enterobacteriaceae* infections whilst reducing the toxic effects of colistin.

## Highlights

- *Mcr* genes are associated with mobile genetic elements that are facilitating its global dissemination.
- Using colistin as a growth promoter increases the risk of acquiring *mcr-*positive *Enterobacteriaceae* in food-producing animals.
- *Mcr* genes have widely disseminated into clinical settings, threatening public health.
- MCR proteins are phosphoethanolamine (PEtN) transferases that mediate the transfer of PEtN from its primary phosphatidylethanolamine to lipid A.
- Multiple compounds can synergistically restore colistin’s activity, reducing its dosage and toxicity.
- CRISPR-Cas9, endolysins-engineered enzymes, and antimicrobial peptides are promising therapies for colistin resistance

## Introduction

Colistin was first introduced into clinical practice in the 1950s (Falagas et al., 2005). It was derived from *Bacillus polymyxa* and belonged to polymyxins (Nigam et al., 2015), a family of cationic polypeptide antibiotics with a narrow-spectrum antimicrobial activity (Ye et al., 2016). Colistin has a bactericidal effect on Gram-negative bacteria and thus is used for treating Gram-negative bacterial infections (Falagas et al., 2005; Nigam et al., 2015). Cationic polypeptides have a high electrostatic attraction to the anionic lipopolysaccharide (LPS) located on the outer membrane of Gram-negative bacteria. There, it displaces the magnesium and calcium divalent cations (Mg^2+^ and Ca^2+^), which stabilize the LPS molecules (Falagas et al., 2005; Nigam et al., 2015). This results in the disruption of the cell’s permeability, leading to cell death. However, due to the adverse side effects such as nephrotoxicity and neurotoxicity seen during colistin therapy (Nigam et al., 2015), in the early 1980s, it was removed from human use and administered to farm-animals as prophylaxis, metaphylaxis, and a therapeutic (Etebu and Arikekpar, 2016). It was further used as a growth promoter in some countries (Dawadi et al., 2021). With the increasing resistance caused by Gram-negative pathogens such as carbapenem-resistant Enterobacteriaceae that threatens global public health (Bulman et al., 2017), colistin was recently reintroduced as a last-line treatment option (Ye et al., 2016; Fyfe et al., 2016; Al Atya et al., 2016).

Colistin resistance was largely associated with chromosomal-encoded mechanisms that involved two-component systems (TCSs) such as PmrAB and PhoPQ, and mutation(s) in the MgrB regulator in *Klebsiella pneumoniae* (Gunn, 2008; Cannatelli et al., 2013; Paterson and Harris, 2016). These chromosomal mutations mediated colistin resistance by modifying LPS, changing LPS’s overall charge, and reducing the affinity of polymyxins to the outer membrane (Gunn, 2008; Cannatelli et al., 2013; Paterson and Harris, 2016). The types of modifications seen include the addition of phosphoethanolamine (PEtN) and 4-amino-4-deoxy-L-arabinose (Ara4N) to the 1-phosphate or 4-phosphate groups of Lipid A, respectively (Mmatli et al., 2020). The PEtN modification is associated with the PmrAB TCSs and the Ara4N modification with the PhoPQ TCS alongside the MgrB regulator (Mmatli et al., 2020).

Plasmid-mediated colistin resistance (*mcr)* gene was first identified in both animals and humans by Liu et al. (2016) from *Enterobacteriaceae*. *Mcr-1* gene encodes a PEtN transferase enzyme that adds a PEtN to Lipid A (Liu et al., 2016) at the 4’-phosphate group, thus inducing colistin resistance (Hu et al., 2016; Hu et al., 2016). PEtN transferases have previously been identified in Gram-negative bacteria, these include the lipooligosaccharide PEtN transferase A (LptA) from *Neisseria meningitides* (Wanty, 2013) and the *Campylobacter jejuni* PEtN transferase, EptC (Wanty et al., 2013; Fage et al., 2014). These PEtN transferases have similar structural properties to the MCR-1 PEtN transferase (Fage 2014). The lipid A modification is identifiable with a matrix-assisted laser desorption/ionization-time of flight (MALDI-TOF) mass spectrometry (MS) assay with an additional *m/z*= 1920.5 peak observed in MCR-producing isolates, representing the modified Lipid A molecule (Xu et al., 2018). This activity is seen across the different *mcr* genes: *mcr-2* to *mcr-10.*

After the discovery of *mcr-1*, other novel *mcr* genes i.e., *mcr-2* to *mcr-10*, which are widely distributed within Enterobacteriaceae, have been reported globally. The identification of the *mcr* genes, *mcr-1* to *mcr-10*, is largely mediated by PCR screening and whole genome sequencing (WGS) tools, which are also used in *mcr* surveillance programmes (Liu et al., 2016; Xavier et al., 2016; Roer et al., 2017; Yang YQ et al., 2017; Rebelo et al., 2018; Wang Y et al., 2020). Each *mcr* gene is mostly located on conjugative plasmids, associated with mobile genetic elements (MGEs), and mediates colistin resistance through PEtN transferase activity. *mcr* are widely distributed within Enterobacteriaceae, including *Escherichia coli, K. pneumoniae, Salmonella* species, and *Enterobacter* species (Ye et al., 2016; Liu et al., 2016; Di Pilato et al., 2016; Anjum et al., 2016), and have also been reported within other Gram-negatives such as *Pseudomonas aeruginosa* and *Acinetobacter* species (Hsu et al., 2017; Snesrud et al., 2018; Snyman et al., 2021).

Initially, food-producing animals were the reservoir of *mcr* genes due to the high usage of colistin in livestock (Liu et al., 2016; Kempf et al., 2016; Monte et al., 2017). However, farmers and employees in slaughterhouses are continuously exposed to livestock and their feces and thus are at risk of acquiring *MCR-*producing isolate (Monte et al., 2017; Runcharoen et al., 2017; Dawadi et al., 2021). Global screening of *mcr-1* genes in livestock found increased number of MCR-producing isolates, resulting in a ban of colistin in food-producing animals for both growth promotion and treatment of bacterial infections in some countries (WHO, 2011; Collignon et al., 2016). The World Health Organisation (WHO), thereafter, listed colistin as part of the critically important antimicrobials for human medicine. This was to help preserve the effectiveness of colistin for clinical use and to minimize the transmission of *mcr* genes from animals, livestock, and the environment to humans (WHO, 2011; Collignon et al., 2016).

### Purpose of review

This review provides a map of the dissemination of *mcr-1* and other *mcr* genes. It further evaluates the genomic content of each *mcr* gene, identifying the possible progenitor and the mobile elements that each is associated with. We largely look at the *mcr-1* gene, its structure and function, which enables it to mediate colistin resistance, the fitness cost imposed by *mcr-1* expression, and the risk factors enabling the dissemination of *mcr* genes. The review further summarizes the possible treatment options for MCR-producing colistin-resistant isolates in humans.

Herein, we highlight the evolution of *mcr* genes by providing insight into the genomic content of each gene, identifying the MGEs that aid in *mcr* dissemination, the global *mcr* distribution, *mcr* crystal structure, and enzymatic activity of MCR proteins. We further discuss promising emerging therapeutics that could manage *mcr-*positive *Enterobacteriaceae* infections.

## Mcr genes genomic context

The *mcr-1* gene is part of a 2,600 base pairs (bp) cassette that is made up of a putative promoter gene responsible for the expression of the *mcr-1* gene and the hypothetical protein later identified as *pap2* (Poirel et al., 2016; Poirel et al., 2017). *Mcr-1* is speculated to have been derived from *Moraxella* species, which harbors the intrinsic chromosomal encoded *mcr*-like genes and the *pap2* membrane-associated lipid phosphatase (Poirel et al., 2017; Kieffer et al., 2017). The *pap2* gene is found in both *mcr-1* and *mcr-2* cassettes and shares 41% identity with *Moraxella oloensis* phosphatidic acid phosphatase (Xavier et al., 2016; Poirel et al., 2017). *Moraxella mcr*-like genes with a significant degree of similarity to *mcr-1* and *mcr-2* in *M*. *porci* and *M*. *osloensis* were respectively identified in Genbank. Thus, these genes could be closely related (Kieffer et al., 2017). Poirel et al. identified an *mcr*-like gene, *mcr-2.2* later labelled *mcr-6*, from an *M*. *pluranimalium* strain with an 82% and 99% amino acid identity to *mcr-1* and *mcr-2*, respectively (Poirel et al., 2017).

An analysis of *mcr-1* sequences deposited in GenBank in 2017 revealed an *mcr-1.10* variant from *Moraxella porci* MSGI3-CO3 with 97.61% identity to the plasmid-borne *mcr-1* gene (AbuOun et al., 2017), additionally an *mcr-2.1* variant 99% amino acid identity to plasmid-born *mcr-2* was identified in *M. pluranimarium* (Poirel et al., 2017). This isolate was isolated from the fecal contents of healthy pigs in the United Kingdom in April, 2014 (Snesrud et al., 2018). This data suggests that *Moraxella* species may have been the likely source of *mcr-1* and *mcr-2* (Poirel et al., 2017; Poirel et al., 2017). Other evidence that supports this speculation that *mcr-1* evolved from the *Moraxella* species is the identification of an IS*Apl1* element in *M*. *bovoculi* and *M*. *porci* (Poirel et al., 2017; Li R et al., 2018). Li et al. suggest IS*Apl1* integrated into *M*. *bovoculi* and thereafter evolved with the *mcr*-like genes to the point seen today (Li R et al., 2018). This synteny of *mcr*-*pap2* genes across *Moraxella* species further highlights this genus as a natural reservoir of *mcr*-like genes and a possible progenitor due to the high amino acid identity (Kieffer et al., 2017; Poirel et al., 2017; AbuOun et al., 2017; Wei W et al., 2018).

To evaluate the phylogenetic relationship between *mcr-1* and *Moraxella* species’ PEA transferase, Wei et al. conducted domain swapping with an intrinsic colistin resistance gene (*icr*) from *M. osloensis* (Icr*-*Mo) (Wei W et al., 2018). PEA transferases are made up of two domains, Transmembrane (TM) and the catalytic domain, discussed later. Thus, Wei et al. created hybrids versions of Icr-Mo gene and found that only TM(MCR-1)-ICR-Mo hybrid had partial activity conferring colistin resistance up to 4 µg/mL and able to modify the Lipid A moiety (Wei W et al., 2018). Therefore, MCR-1 and ICR-Mo are not fully interchangeable, which indicates a fundamental difference between the two proteins (Wei W et al., 2018).

The mobilization of the *mcr*-*pap2* unit was thereafter accomplished through IS elements, but Kieffer et al. identified a replicase gene associated with *mcr-1* on IncX_4_ plasmids (Kieffer et al., 2017). The gene had a 99% identity to *M*. *lacunata*, thus showing this species as a possible reservoir of IncX_4_ plasmids and further speculating that the *Moraxella* family may encode genetic tools likely involved in the initial mobilization of *mcr* genes (Kieffer et al., 2017).

Stoesser et al. and Sun et al. suggested that the initial mobilization of *mcr-1* genes into *Enterobacteriaceae* was IS*1294*-mediated, using a one-ended rolling circle transposition mechanism shown to be capable of mobilizing adjacent sequences (Stoesser et al., 2016; Sun et al., 2018). The IS*1294* may have mobilized the *mcr-1* cassette into an IS*Apl1* composite transposon, creating an IS*Apl1*-*mcr-1*-*pap2*-IS*1294*-*pap2*-IS*Apl1* cassette, which has been identified on *E. coli* chromosome (Stoesser et al., 2016; Sun et al., 2018). It was found that the cassette was still flexible enough to jump from the chromosome to a plasmid (Sun et al., 2018) by generating a putative circular intermediate product.

**Figure 1:**
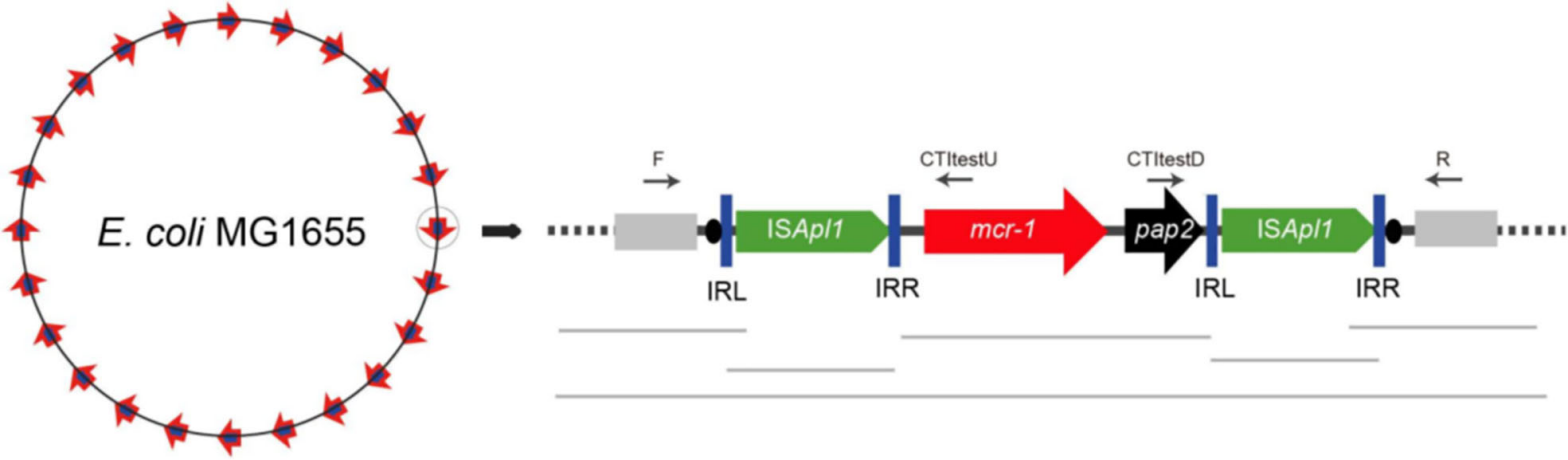
A schematic diagram of the ISApl1 composite transposon identified on the Escherichia coli chromosome retrieved from He et al. (2019). The diagram is made up of mcr-1 gene in red, the hypothetical pap2 protein in black, the inverted repeats (IRL and IRR) in blue vertical bars and the direct repeats as black ovals.

There is increasing evidence, however, that the *mcr-1* gene is mobilized primarily as a composite transposon, Tn*6330*, that is made up of two copies of IS*Apl1* that bracket cassettes (**Figure 1**) (Snesrud et al., 2016; Zurfluh et al., 2016). IS*Apl1* is an IS that was first described in *Acinetobacillus pleuropneumoniae* and is part of the IS*30* family (Tegetmeyer et al., 2008). The IS elements of this family are flanked by 20-30-bp inverted repeats (left IR (IRL), right IR (IRR)), which are essential for transposition (Szabó et al., 2008). The IR contains a 924 bp open-reading frame that encodes a 44.3 kDa transposase protein containing a DDE domain, which encodes three conserved amino acid residues viz., D_228_, D_295_, and E_648_ (DDE), as well as carboxylase residues that help coordinate metal ions for catalysis (Szabó et al., 2008; Snesrud et al., 2016). Analysis of each IS*Apl1* element flanking the *mcr-1*-*pap2* unit found conserved dinucleotides between the IS*Apl1* inverted repeats and the *mcr-1*-*pap2* unit, an AT dinucleotide on the IRR of the upstream element and CG on the IRL of the downstream element (Snesrud et al., 2016) (**Figure 1**). An interesting observation in the *mcr-1.10* variant identified in *Moraxella* sp. MSGI3-CO3 was the presence of these dinucleotides, AT upstream and CG downstream, flanking the *mcr-1* structure (Snesrud et al., 2018). These dinucleotides were suggested to represent the ancestral target-site duplications (TSDs) formed during the initial mobilization of *mcr-1* during IS*Apl1* insertion (Snesrud et al., 2018). Therefore, Snesrud et al. suggested that the formation of the composite transposon, Tn*6330*, was through two independent insertion events of IS*Apl1* into the TA-rich region of the *mcr-1*-*pap2* unit, generating the conserved interior 2bp TSDs, AT and CG (Snesrud et al., 2018). Subsequent transposition of the Tn*6330* would therefore generate new TSDs at the new target site but would retain both internal conserved 2bp dinucleotides (Snesrud et al., 2018).

As stated above, the IS*Apl1* is part of the IS*30* family. The family has been shown to mobilize through a copy (out) and paste mechanism, forming circular intermediates of a single IS during transposition. The family is further known to have a high affinity for certain target sites resembling their IR sequence (Tegetmeyer et al., 2008; Szabó et al., 2008; Wang Q et al., 2018). Multiple studies have investigated the mechanisms of IS*Apl1* in mobilizing the *mcr-1* gene and found that during each transposition event, the transposon was a circular intermediate covalently closed doubled stranded DNA, 5 699 bp in size and generated a 2 bp direct repeats at the insertion site which was an AT-rich region (Szabó et al., 2008; Snesrud et al., 2016; Poirel et al., 2017; Snesrud et al., 2017), a similar mechanism seen in the IS*30* family. IS*Apl1* is most likely an important factor responsible for the insertion and fixation of the *mcr-1* gene into various classes of self-transmissible plasmids and host chromosomes (Poirel et al., 2017; Zhao et al., 2017; Li R et al., 2018). The formation of an intermediate structure consisting of IS*Apl1* and *mcr-1* during mobilization indicates that the resistance genes have become highly mobilizable in both plasmids and the chromosomes (Li R et al., 2018). After the first identification of *mcr-1* in pHNSHP45 (Liu et al., 2016), an Incl_2_ plasmid, *mcr-1* was thereafter detected in a wide range of conjugative plasmids, IncI_2_, IncHI_2_, IncX_4_, IncF, and IncP with the potential to mediate the dissemination of *mcr-1* genes into other Gram-negative bacteria (Zhao et al., 2017). Petrillo et al. suggest that the insertion of the complete transposon triggers the rapid mobilization of conjugative plasmids, encouraging their dissemination across the Enterobacteriaceae family (Petrillo et al., 2016). The copy out and paste in mechanisms allow for the transposition of the *mcr-1* cassette, and the decay properties of IS*Apl1* further transfix the resistance gene into a plasmid or chromosome (Snesrud et al., 2017; Sia et al., 2020).

**Figure 2:**
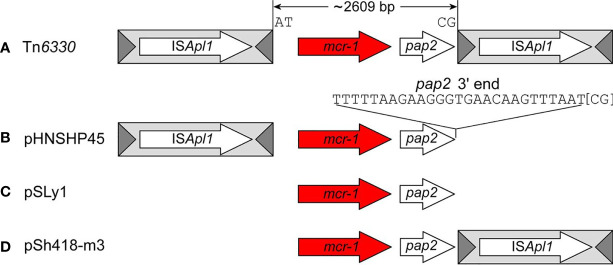
The four general structures of mcr-1 sequences created by the deletion events retrieved from Snesrud et al. (2018). The structures include **(A)** the complete composite transposon with both copies of ISApl1 elements (Tn6330), **(B)** Structures with a single copy of ISApl1 located upstream of mcr-1 (pHNSHP45); **(C)** structures that lost both copies (pSLy1) and **(D)** a fourth structure with a single copy of ISApl1 located downstream (pSh418-m3).

Snesrud et al. and Li et al. discovered that IS*Apl1* is highly active and that a single copy of IS*Apl1* can mobilize independently of *mcr-1* across the host genome in AT regions with a slight central GC bias (Snesrud et al., 2017; Li et al., 2019). A sequencing analysis of four *mcr-1* containing isolates performed by Snesrud et al. identified two to six copies of IS*Apl1* element throughout the isolates’ genome. The highly active nature of the IS*Apl1* elements thereafter triggers the deletion of the flanking IS*Apl1* copies to prevent further plasmid rearrangements (Snesrud et al., 2017; Snesrud et al., 2018). Snesrud et al. analysis of the *mcr-1* sequence environment showed that Tn*6330* has a strong tendency to decay through deletion, removing parts of, or both copies of IS*Apl1*, thus transfixing *mcr-1* into a vector plasmid (**Figure 2**). This has led to the observation of many sequences lacking one or both of IS*Apl1* (Snesrud et al., 2016; Poirel et al., 2017). Composite transposons in the IS*30* family have been shown to contribute to replicon stabilization through transposition and illegitimate recombination (Szabó et al., 2008; Snesrud et al., 2016). The loss of IS*Apl1* elements results in the loss of transposability, stabilizing the *mcr-1* cassette in plasmids, which facilitates the widespread dissemination of the colistin resistance gene in self-transmissible plasmids (Snesrud et al., 2016; Tarabai et al., 2019; Ji et al., 2019). As discussed, IS*Apl1* has a significant bias for insertion in AT-regions and generates TSD of two or three bases. The analysis of the *mcr-1* cassette in the absence of IS*Apl1* elements has shown that the cassette is found in similar locations as per plasmid type and flanked by conserved trinucleotides (5’-ATA-3’) that are found immediately downstream of the IS*Apl1* IRR (Snesrud et al., 2016; Tijet et al., 2017). This is because the deletion event involves one to four flanking nucleotides that remain at the deletion junction (Snesrud et al., 2018).

An analysis of *mcr-1* sequences deposited in the public database has shown four general structures of *mcr-1* sequences: the complete composite transposon with both copies of IS*Apl1* elements; structures with a single copy of IS*Apl1* located downstream of *mcr-1*; structures that lost both copies and a rare fourth structure with a single copy of IS*Apl1* located upstream (Snesrud et al., 2016; Snesrud et al., 2018) (**Figure 2**). In structures with a single copy of IS*Apl1* found upstream and the IRR sequence of the downstream of deleted IS*Apl1*, Snesrud et al. suggest that the transposase encoded by the upstream IS*Apl1* can recognize the downstream IRR and thereafter still be able to mobilize the bracketed region without a complete composite transposon (Snesrud et al., 2016). The partially or complete removal of IS*Apl1* was through an illegitimate recombination that generated mismatches and deletions. Sun et al. found that the 3’ end of the *mcr* cassette unit was flexible in all IncX_4_ plasmids and the sequence could match with the perfect IRR of IS*Apl1*, though all IncX_4_ currently lack IS*Apl1* elements (Sun et al., 2018). The evidence from this study suggests that the TSD generated, and the six mismatches acquired through illegitimate recombination could be identified as a “relic” to track an insertion event that resulted in the subsequent loss of IS*Apl1* (Sun et al., 2018). The loss of IS*Apl1* elements in IncX_4_ was conducive to maintaining the *mcr-1* cassette on the plasmid (Sun et al., 2018), increasing its stability, and thus allowing for the dissemination of resistance genes *via* the plasmids (Snesrud et al., 2016; Tarabai et al., 2019; Ji et al., 2019). The identification of these IRR in IncX_4_ plasmids allow for the conclusion that IS*Apl1* was associated with the transposition of the *mcr-1* cassette into IncX_4_ plasmids (Sun et al., 2017a).

**Figure 3:**
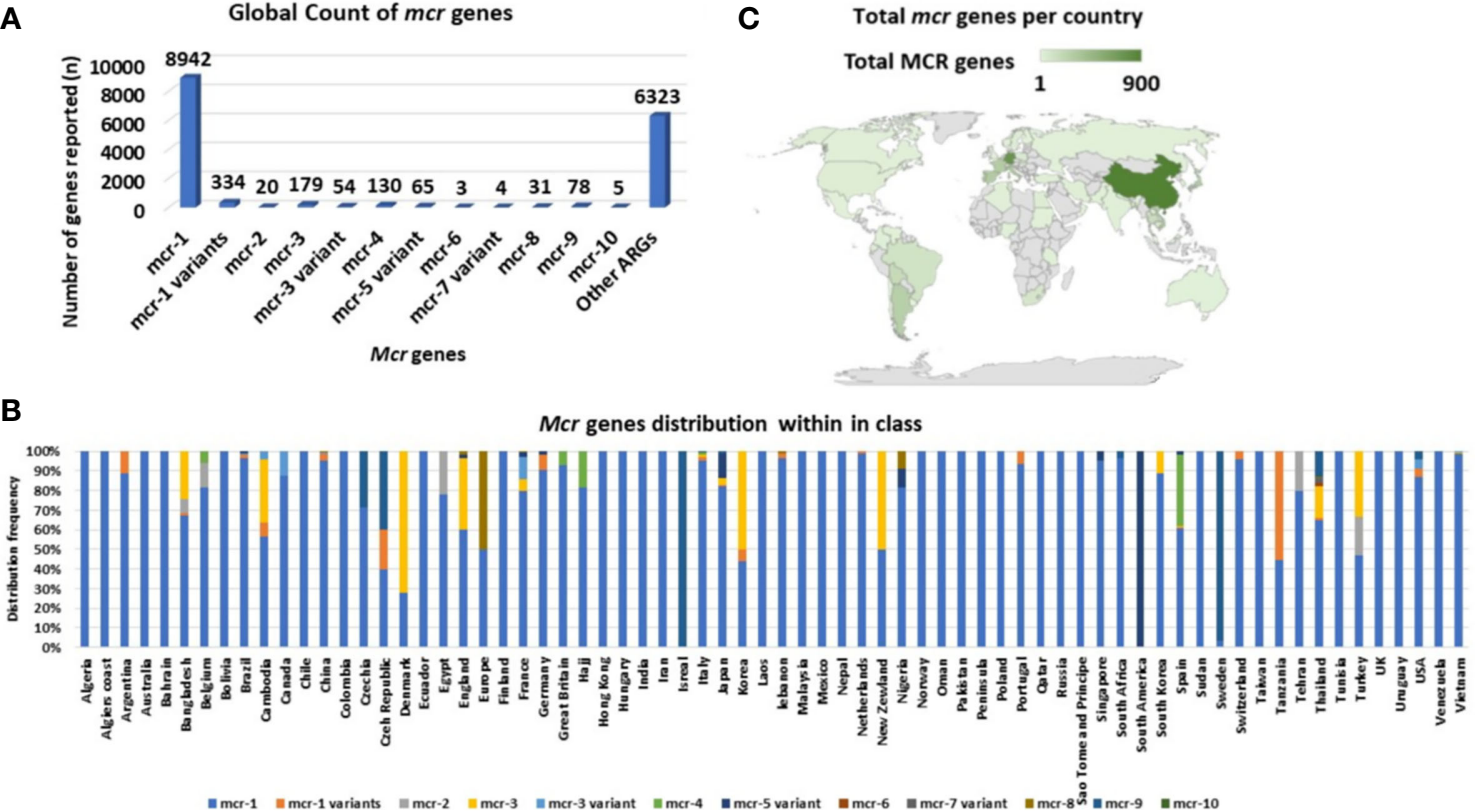
Global distribution and total count of mcr genes. **(A)** Total number of mcr genes reported globally. **(B)** The distribution of mcr genes per country. **(C)** Global map showing the geographical distribution of mcr genes.

**Figure 4:**
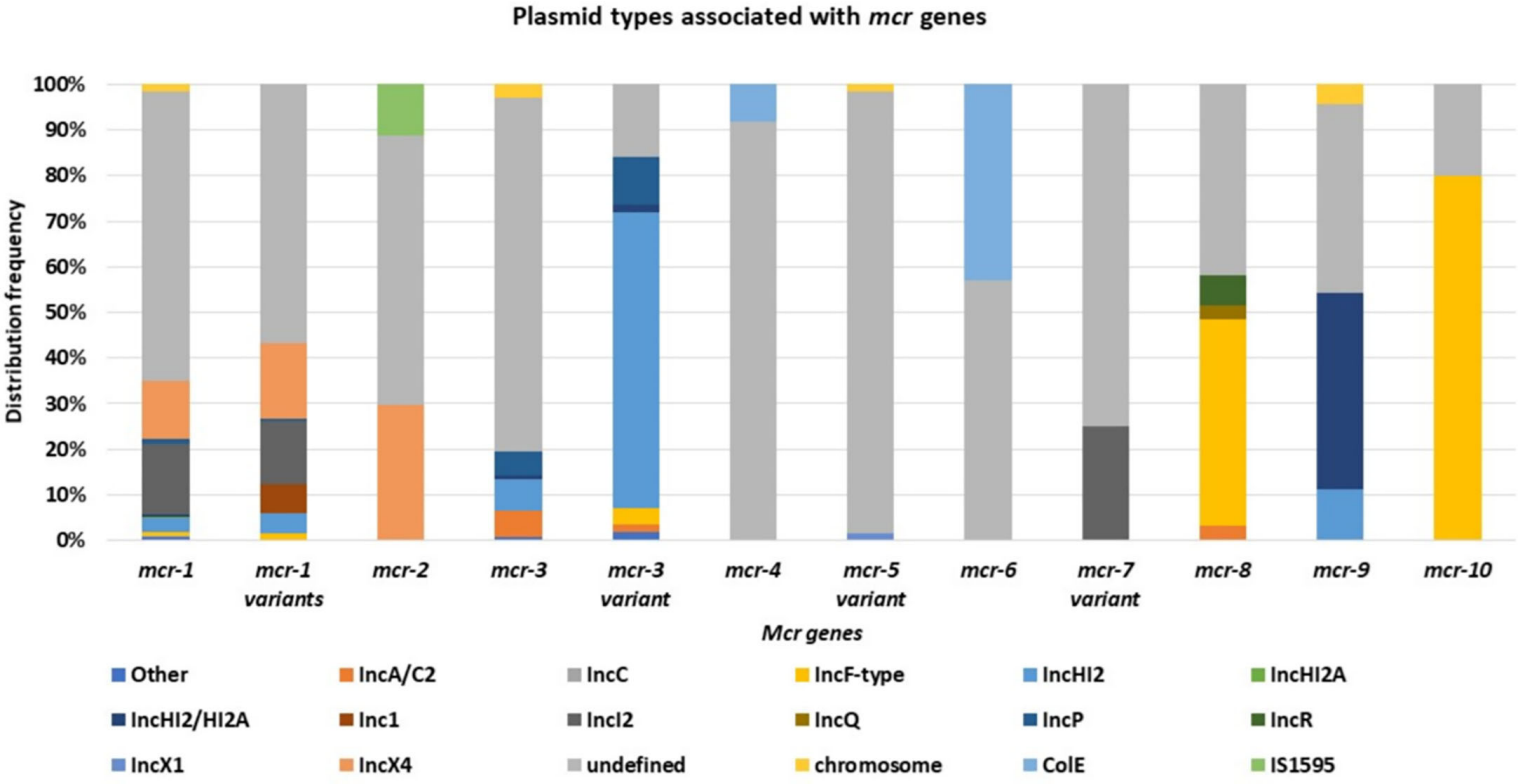
The distribution of plasmid incompatibility groups associated with each *mcr* gene.

*Mcr-1* and its variants (*mcr-1* variants) are the most common and well disseminated of all *mcr* genes (**Figure 3**). This was later shown to be because of multiple factors such as fitness cost and MGEs. They have spread globally and have been identified in 69 countries, with *mcr-1* being the most prevalent *mcr* gene in most countries (**Figure 3**). Although the other *mcr-*genes have spread globally, they have been identified in low numbers compared to *mcr-1* (**Figure 3A**). *Mcr-1* and its variants have been identified to be associated with 37 different plasmid incompatibility groups, with IncI_2_, IncX_4_, IncHI_2_, and IncP being the most prevalent (**Figure 4**, **Table S6**). These incompatibility groups have also been shown to be broad-spectrum plasmids which further harbor other resistance genes.

The *mcr-2* was first identified in ten *E. coli* isolates during a passive surveillance screening of diarrhea in calves and piglets, and like *mcr-1*, *mcr-2* was located on a conjugative plasmid (IncX_4_) and was able to confer clinical colistin resistance (4-8 mg/L) (Xavier et al., 2016). The gene was identified due to the presence of a putative membrane protein identified as a PEtN transferase in *mcr-1* negative isolates (Xavier et al., 2016). *Mcr-*2 has a 76.75% and 80.65% nucleotide sequence and amino acid identity, respectively, to *mcr-1* (Xavier et al., 2016).

A genetic analysis of the IncX_4_ plasmid found that *mcr-2* was located within two IS*1595*-like ISs and a 279 bp open reading frame (ORF), located downstream the *mcr-2* gene (Xavier et al., 2016). The ORF encodes a PAP2 membrane-associated lipid phosphatase which is related to the PAP2 protein encoded by the ORF of *mcr-1* (Xavier et al., 2016; Partridge, 2017). The ORF of *mcr-2* has a 41% identity to the phosphatidic acid phosphatase encoded by *M*. *osloensis*. When Xavier et al. removed the *mcr-2*-*pap2* from the IS*1595* backbone, a BLASTn search produced a single hit to *M*. *bovoculi* strain with a 75% identity across a 100% query coverage (Xavier et al., 2016).

The IS*1595* element is composed of a transposase gene flanked by two inverted repeats (18 bp each). *Mcr-2* is thus present within a complete transposon which allows for horizontal gene transfer (Sun et al., 2017b). Sun et al. found that the IS*1595* composite transposon was able to form circularized intermediates, which were identified during an inverse PCR assay (Sun et al., 2017b). Hence, the IS*1595* elements are involved in the mobilization of the *mcr-2* cassette *via* homologous recombination events, which like *mcr-1*, allows for the dissemination of the resistance gene across diversified bacterial hosts (Sun et al., 2017b).

After the first identification of *mcr-2* in Belgium on an IncX_4_ plasmid, an effective vehicle for dissemination of resistance and virulence genes with a high transfer frequency (Xavier et al., 2016; Sun et al., 2017b), it was thereafter identified in *Salmonella* sp. on IncX_4_ plasmids in Belgium in 2018 from retail meat collected in 2012 (Garcia-Graells et al., 2018). It has subsequently been found in pig and poultry samples in 19 provinces in China, where the prevalence of *mcr-2* in the study was 56.3% in poultry (Zhang et al., 2018). *Mcr-2* was then found in clinical settings in Iran, in stool samples collected between 2011-2016 (Ghasemian et al., 2019). In Egypt, *mcr-2* was widespread and identified in humans, wild birds, and the environment (water sources) (Ahmed et al., 2019). Compared to *mcr-1*, *mcr-2* has not spread across the world (**Figure 3A**). and imposed a big threat to public health though found on a low fitness burden plasmid, IncX4 (**Figure 4**). The gene has only been identified in Bangladesh, Belgium, and Egypt in low numbers (**Figure 3B**). The gene has however spread well within the Enterobacteriaceae family i.e., *E. coli*, *K. oxytoca*, *K. pneumoniae* and *Salmonella* sp.

AbuOun et al. (2017) identified an *mcr-2.2* variant in *Moraxella pluranimalium* strain isolated from a pig in Great Britain. The MCR-2.2 protein was found to have an 87.9% amino acid identity to MCR-2, encoding 65 amino acid substitutions (AbuOun et al., 2017). Partridge et al. (2018) suggested that since *mcr-2.2* encodes a protein that is 87.9% identical to the original MCR-2, it should be labelled as *mcr-6*. The *mcr-6* gene like *mcr-1* and *mcr-2* was associated with the PAP2 gene located downstream of *mcr-6* (AbuOun et al., 2017). The *mcr-6* has only been identified in the *M*. *pluranimalium* MSG47-C17 strain isolated in 2012 and has not disseminated into the Enterobacteriaceae family, identified only in *E. coli*. AbuOun et al. (2017) however highlights that *Moraxella* species could have been the source of *mcr*-*pap2* genes.

The first identification of *mcr-3* was in *E. coli* isolated from pigs in China (Yin et al., 2017). Thereafter, multiple *mcr-3* variants have been identified with one or more amino acid substitutions. *Mcr-3* genetic context is made up of a diacylglycerol kinase (*dgkA*) gene located downstream the *mcr-3* gene and is thereafter flanked by a truncated (Δ) IS*Kpn40* upstream and an intact IS*Kpn40* downstream (Sia et al., 2020). In some cases, a ΔTn*As2* element is located upstream of the ΔIS*Kpn40* element (Yin et al., 2017; Liu et al., 2017; Sia et al., 2020), and the genetic context (ΔTn*As2*-ΔIS*Kpn40*-*mcr-3*-*dgkA*-IS*Kpn40*) is flanked by an IS*26* element upstream and an intact IS*15DI* element downstream (Creighton et al., 2019; Gomi et al., 2019; Sia et al., 2020). These elements, IS*Kpn40*, Tn*As2*, IS*26* and IS*15DI* were identified and hypothesized to play a role in the transposition of the *mcr-3* cassette among different species or bacterial genera (Wang et al., 2018; Xiang et al., 2018; Sia et al., 2020). Sia et al. identified two circular intermediates of 3535 bp and 5990 bp made up of *mcr-3*-*dgkA*-IS*Kpn40* and ISΔ*26*-Tn*As2*-ΔIS*Kpn40*-*mcr-3*-*dgkA*-IS*Kpn40*, respectively, during conjugation experiments (Sia et al., 2020). Wang et al. evaluated the transferability of the 5990 bp circular intermediate and concluded that the ΔIS*26* and intact IS*15DI* mobilises the *mcr-3.1 via* homologous recombination through the formation of circular intermediates (Wang et al., 2018). The IS-mediated transposition enables the mobilization of the *mcr-3* resistance gene between the chromosome and plasmids, which further contributes to the dissemination of resistance genes (Wang et al., 2018; Gomi et al., 2019). Similar conclusion was reached with the 3535 bp circular intermediates in a study performed by Xiang et al., which showed that mobilization of the *mcr-3* core segment (*mcr-3*-*dgkA*) through IS*Kpn40* elements facilitated its spread into various plasmids (Xiang et al., 2018).

The *mcr-3.12* variant has a 99% nucleotide sequence identity with an *Aeromonas veronii* sequence (Kieffer et al., 2018) and significant (77%) amino acid sequence identity to three PEtN transferases found within the genera (Yin et al., 2017; Cabello et al., 2017; Kieffer et al., 2018). It is suggested that *mcr-3* genes originated from the *Aeromonas* genus, or the *mcr-3* gene is widely disseminated as an acquired resistance trait and thus also found in Aeromonads (Kieffer et al., 2018). It is however thought that *mcr-3* was initially transposed from the *Aeromonas* genera to the Enterobacteriaceae family through IS*Kpn40*-mediated transposition. The IS*Kpn40* elements encode two ORF and the second ORF has a 99% identity to both a transposase from *Aeromonas* sp. and an integrase from *Aeromonas caviae* (Wang Z et al., 2019). This IS element is found flanking the *mcr-3* segment and was also shown to mobilize through homologous recombination (Kieffer et al., 2018; Sia et al., 2020). The last evidence of *mcr-3* originating from *Aeromonas* includes the presence of Tn*As2* located upstream of the *mcr-3* gene (Yin et al., 2017). The transposon has only been identified in *A. salmonicida* and because its sequence in pWJ1 (original *mcr-3* plasmid) is a partial sequence, it is unlikely to have mobilized the *mcr-3* gene (Yin et al., 2017; Liu et al., 2017). This evidence supports the possibility of the *Aeromonas* genus being a possible progenitor of *mcr-3* genes (Yin et al., 2017).

*Mcr-3* and its variants are disseminated within Enterobacteriaceae, having been identified in *E. coli*, *Salmonella* sp. and *K. pneumoniae* in healthcare centers, aquaculture, wastewater, and in animals (Roer et al., 2017; Yin et al., 2017; Liu et al., 2017; Cabello et al., 2017; Litrup et al., 2017; Wang et al., 2018a; Gomi et al., 2019). They have further been identified in eighteen countries, with Colombia and Thailand having the highest counts (**Table S3**, **Figure 3C**). The resistance genes have been identified in both transmissible and non-transmissible plasmids such as IncA/C_2_, IncHI_2_, IncHI_2A_, IncFII/FIB, IncY, IncR, IncF, and IncP and in many variant mutations with IncHI_2_ being the most prevalent (**Table S6**, **Figure 4**).

*Mcr-4* was first identified in *S.* Typhimurium isolated from the cecal content of a pig at slaughter in Italy in 2013 (Carattoli et al., 2017; Carattoli et al., 2018). The *S.* Typhimurium was colistin-resistant and negative for known *mcr* genes; the *mcr-4* gene was identified through whole genome sequencing (Carattoli et al., 2017). MCR-4 respectively has an amino acid sequence identity of 34%, 35% and 49% to MCR-1, MCR-2, and MCR-3, and like other PEtN transferases, can mediate colistin resistance through Lipid A modification (Carattoli et al., 2017). *Mcr-3* and *mcr-4* are suggested to have originated from aquatic environments, as *Aeromonas* and *Shewanella* sp. are aquaculture fish pathogens that are intrinsically resistant to colistin (Cabello et al., 2017). MCR-4 has an 82%-99% amino acid sequence identity to one large PEtN transferase found in *Shewanella* sp., and the *mcr-4.3* variant has a 100% nucleotide sequence identity to a chromosomal PEtN transferase encoded by *S. frigidimarina*, Sfri_3478 (Cabello et al., 2017; Carattoli et al., 2017; Chavda et al., 2018). Zhang et al. suggests a horizontal transfer of *mcr-4.3* from *S. frigidimarina* into a diversified species of Enterobacteriaceae (Zhang et al., 2019).

The *Shewanella* genus encodes eight variants of PEA transferases called non-mobile colistin resistance genes (*nmcr*) that confer colistin resistance of up to 16 µg/mL (Zhang et al., 2019; Zhang et al., 2019a). Zhang et al. suggests the intrinsic *nmcr-1* enzyme, Sfri_3478, as a progenitor of *mcr-4* rather than *mcr-1/2* enzymes. Zhang et al. further evaluated the interdomain communication between *mcr-4* and *nmcr-1* using the domain swapping method (Sun et al., 2017b). The expression of TM(NMCR-1)-MCR-4 and TM(MCR-4)-NMCR-1 in the recipient strain of *E. coli* MG1655 conferred resistance to colistin up to 8 µg/mL and resulted in LPA modification by an addition of PEA. Thus, the chimeric versions of NMCR-1/MCR-4 are functional in their catalytic activities (Zhang et al., 2019a).With this evidence, *Shewanella* genus may be a progenitor of MCR-4 and there may be diverse evolutional paths across the entire family of MCR-1 enzymes (Zhang et al., 2019; Zhang et al., 2019a).

Though *mcr-4.3* encodes two-point mutations of C179G and V236F which inactivate the PEA transferase activity, Zhang et al. shows that progressive evolution can partially restore its action (Zhang et al., 2019a). This was performed by creating two revertants of *mcr-4.3* (G179V and F236V, respectively), each revertant conferred colistin resistance of up to 8 µg/mL and resulted in Lipid A modification (Zhang et al., 2019a).

*Mcr-4* was initially identified on an 8749 bp ColE10 plasmid, which encoded a RepB replicase, mobA/L mobilization proteins, a RelE toxin and excl1 gene (Carattoli et al., 2017; Carattoli et al., 2018). In other cases, the ColE plasmid was found to encode a RepA replicase instead of RepB. However, both plasmids showed a 99% nucleotide sequence identity to plasmids from *Pantoea* species, with a 65% coverage (Carattoli et al., 2017; Chavda et al., 2018; García et al., 2018). The *mcr-4* gene was flanked by IS*5* elements on some plasmids and on other plasmids, it was flanked by an ΔIS*10* element upstream and an IS*1294* element downstream (García et al., 2018). Genetic analysis found that the ColE plasmid encodes a conserved 59-bp region upstream the *mcr-4* gene, and this region is predicted to encode the -35 (TTATTT) and -10 (AGCTAGTAT) promoter regions (Chavda et al., 2018). This allows for ColE plasmids to replicate independently in different bacterial species and genera (Carattoli et al., 2017). ColE plasmids are broad-range non-conjugative plasmids whose mobilization, however, require a helper plasmid; nevertheless, the mobilization of the *mcr-4* gene is hypothesized to be achieved through a transposition event mediated by the IS*5* element (Carattoli et al., 2017).

A genomic analysis of the initial *mcr-4*-producing *S.* Typhimurium genome found the ColE10 replicon located within the host chromosome integrated within the Type 1 methylation gene. Carattoli et al. suggest a transposition event mediated the chromosomal integration of the ColE10 plasmid (Carattoli et al., 2017). *Mcr-4* has been isolated from both humans and animals in Spain, Belgium, Great Britain, Hajj Italy, and China (**Figure 3C** and **Table S3**) in *E. cloacae, E. coli, L. adecarboxylata and, S. enterica* (**Table S3**) (Carattoli et al., 2017; García et al., 2018; Teo et al., 2018; Sun et al., 2019).

*Mcr-5* was first identified in a d-tartrate-fermenting *S*. Paratyphi isolated from food-producing animals across Germany between 2011 and 2013. Following this discovery of *mcr-5*, further screening resulted in the identification of fourteen additional *mcr-5*-producing isolates. These were isolated between 2011 to 2016 in the same study (Borowiak et al., 2017).

Genetic analysis of *mcr*-5 found that the resistance gene is located within an operon encoding a Chromate B (ChrB) protein domain responsible for regulating the expression of ChrA for chromate resistance and two ORF that encode a major facilitator superfamily (MFS) type transporter (Borowiak et al., 2017; Fleres et al., 2019). The complete operon is located within a 7337bp Tn-3 type transposon named Tn*6452* (Borowiak et al., 2017; Sia et al., 2020), that is flanked by inverted repeats (IRs). A genetic analysis of the transposon identified *tnpA* encoding a Tn*3* transposon, *tnpR* encoding a Tn*3* resolvase and a *bla* gene encoding a β-lactamase (Wang et al., 2018b; Guo et al., 2019; Zhang et al., 2019b). Tn*6452* encodes mechanisms for transposition of the *mcr-5* cassette, TnpA, and TnpR (Guo et al., 2019), which allows the *mcr-5* cassette to be transferred between plasmids and chromosomes (Borowiak et al., 2019).

*Mcr-5* has also been identified on Tn*3*-like structures that encode the ChrB gene but lacked the transposase gene and encoded an imperfect Tn*3*-like inverted repeats left (IRL) (Hammerl et al., 2018; Borowiak et al., 2019; Kieffer et al., 2019). This structure resembles features of a miniature inverted repeat transposable element, which possess a left or right IRs but lacks a transposase gene (Kieffer et al., 2019). Kieffer et al. suggests the acquisition of the *mcr-5* cassette was through a transposition event (Kieffer et al., 2019) and although the Tn*3*-like structure lacks a transposase gene, it can still mobilize the cassette through a nonautonomous transposition mechanism (Kieffer et al., 2019). The *mcr-5* cassette has been identified on the host chromosome (Borowiak et al., 2017), a multicopy ColE-type plasmid (Borowiak et al., 2017; Hammerl et al., 2018; Ma et al., 2018; Borowiak et al., 2019) and on an IncX plasmid (Borowiak et al., 2019). The presence of *mcr-5* cassette on a multicopy plasmid (ColE-type) thus means multiple copies of *mcr-5* transposon will exist within the cell. Borowiak et al. found that there is a higher degree of colistin resistance (8 mg/L) in isolates harboring ColE-type plasmids encoding *mcr-5* than in isolates with a single copy of the *mcr-5* integrated within the chromosome (4 mg/L) (Borowiak et al., 2017). The ColE type plasmids are, however, non-conjugative and thus the presence of the *mcr-5* cassette on an IncX plasmid allows for horizontal transfer and dissemination of resistance gene between different bacterial genera and species (Guo et al., 2019; Kieffer et al., 2019). *Mcr-5* genes are not well disseminated within the Enterobacteriaceae family, identified only in *E. coli* and *S.* Typhimurium (**Table S4**) however these genes have been reported in 10 countries, with Japan, Germany and France being the most prevalent (**Table S3**)

*Mcr-7.1* was first identified in chicken in China (Yang et al., 2018). It has thereafter been identified in environmental samples (Furlan et al., 2020a; Furlan et al., 2020b) and fecal samples in Brazil (Dos Santos et al., 2020). The putative PEtN transferase has a 78% nucleotide sequence identity to part of the *mcr-3* gene (Yang et al., 2018). Genetic analysis of *mcr-7.1* identified a 381 bp ORF encoding the diacylglycerol kinase, *dgkA*, downstream of *mcr-7.1*. The *dgkA* has an 82% nucleotide sequence identity to *Aeromonas* (Yang et al., 2018). Yang et al. suggests, like *mcr-3*, *mcr-7.1* originates from *Aeromonas* (Yang et al., 2018). This was derived based on the close genetic distance between MCR-3 and MCR-7.1 found during a phylogenetic analysis and the presence of *dgkA* gene downstream both *mcr-3* and *mcr-7.1* (Yang et al., 2018). The ORF sequence found upstream the *mcr-7.1* gene has an 81% nucleotide sequence identity to a putative phosphodiesterase gene found in *Aeromonas* (Yang et al., 2018). This evidence suggests that the *Aeromonas* genus is a possible progenitor of *mcr-7.1*.

*Mcr-7.1* was identified on a self-transmissible IncI_2_ type plasmid with no ISs in its environment; hence, the dissemination of *mcr-7.1* may be achieved through plasmid mobilization (Yang et al., 2018). *Mcr-7* and its variants, however, are not well disseminated, reported only in three countries, Brazil, China and Thailand (**Table S3**), identified only in two Enterobacteriaceae species, *E. coli* and *K. pneumoniae* (**Table S4**).

*Mcr-8* was initially identified in *K. pneumoniae* from a swine fecal sample in China (Wang et al., 2018b). Conjugation assays found that it is a functional PEtN transferase, and its expression resulted in a four- and eight-fold increase in colistin MIC (Wang et al., 2018b; Wang X et al., 2019; Bonnin et al., 2020). MCR-8 mediated PEtN modification, which was identified using a MALDI-TOF-based technique that discriminates between colistin resistance mechanisms, i.e *mcr*-expression or chromosomal-mutations (Bonnin et al., 2020). BLASTn analysis showed that *mcr-8* has a 50.23% nucleotide and 39.96% amino acid sequence identity to part of *mcr-3* but has 30% to 40% amino acid sequence identity to other MCR proteins.

Ullah et al. performed an *in-silico* search using an MCR-8 probe to detect putative chromosomally encoded PEA transferases (Ullah et al., 2021). The search identified a *nmcr-2* PEA transferases in the plant pathogen *Kosakonia pseudosacchari* with a 67.3% identity to MCR-8. Ullah et al. further showed that NMCR-2 is a possible progenitor of MCR-8 by evaluating their protein engineering, physiological impact, and biochemical analysis (Ullah et al., 2021). The biochemical analysis found that the functional expression of both *mcr-8* and *nmcr-2* in *E. coli* conferred the same level of resistance and physiological impact i.e., retarded growth was observed for expression of both PEA transferase (Ullah et al., 2021). The two PEA transferases were further shown to be domain compatible using the domain-swapping method (Sun et al., 2017b), which showed that the two domains of *nmcr-2* were compatible and functionally exchangeable with *mcr-8* counterparts (Ullah et al., 2021). This validates the phylogenetic position of *nmcr-2* and *mcr-8*.

Analysis of *mcr-8*’s genetic environment found that it is usually flanked by two complete IS*903* elements, with both 50 bp IRL and IRR located upstream, and downstream, respectively (Wang X et al., 2019; Wu et al., 2020). It is also associated with IS*Ecl1* and IS*Kpn26*, located upstream and downstream, respectively (Yang et al., 2019). Yang et al. found that the insertion of IS*Ecl7* was an independent event and may not be involved in mobilizing *mcr-8* (Yang et al., 2019). The formation of circular intermediates *via* IS*903B* elements to mobilize *mcr-8* remains unknown; however, the association of *mcr-8* to ISs facilitates both the transmission of *mcr-8* and its close association with other resistance genes (Wu et al., 2020); specifically, it has been found associated with aminoglycoside resistance genes (Wu et al., 2020), β-lactamase genes (Wang X et al., 2019) and *tmexCD1-toprJ1* genes that encode a novel plasmid-mediated efflux pump that confers resistance to tigecycline (Sun et al., 2020).

*Mcr-8* is commonly found in *K. pneumoniae* (Wang et al., 2018b; Bonnin et al., 2020; Wu et al., 2020; Sun et al., 2020; Salloum et al., 2020; Ngbede et al., 2020; Nabti et al., 2020), Wang et al. performed a phylogenetic analysis of the *mcr*-positive *K. pneumoniae* isolates identified in their study and found that the *mcr*-positive *K. pneumoniae* were genetically diverse (Wang et al., 2018b). K*. pneumoniae* express colistin resistance usually through chromosomal mutations (Mmatli et al., 2020). Wu et al. suggest the expression of *mcr-8* may have synergistic effects with the chromosomal mutations within the TCS, resulting in heterogenous colistin resistance mechanisms (Wu et al., 2020). *Mcr-8* has also been identified in *Klebsiella quasipneumoniae* (Yang et al., 2019) and *R*. *ornithinolytica* as well as on a diverse range of Inc groups: IncFII, IncQ, IncR, IncFIIK, IncFIA and IncA/C (**Table S5**); these will expectedly accelerate the dissemination of *mcr-8* and other ARGs within and outside Enterobacteriaceae (Wang X et al., 2019; Wu et al., 2020). *Mcr -8* genes have reported in seven countries with China being the most prevalent (**Table S3**) and have been identified in four Enterobacteriaceae species, *K. pneumoniae, K. quasipneumoniae, R. ornithinolytica and E. coli.*

*Mcr-9* gene was identified by Carroll et al. (2019) in a colistin susceptible *S.* Typhimurium isolated from a human patient in Washington State in 2010. Further, the *mcr-9* gene was found associated with *wbuC*, encoding a cupin fold metalloprotein, located downstream the gene (Yuan et al., 2019; Kieffer et al., 2019; Osei Sekyere et al., 2020). The presence of *mcr-9* on a plasmid replicon facilitates its dissemination globally and across Enterobacteriaceae (Carroll et al., 2019). It has thereafter been identified in more clinical settings (Chavda et al., 2019; Yuan et al., 2019; Kananizadeh et al., 2020; Lin et al., 2020; Ngbede et al., 2020; Osei Sekyere et al., 2020; Ding et al., 2021; Ribeiro et al., 2021) and in food-producing animals (Cha et al., 2020; Khanawapee et al., 2021). Among the 355 genomes identified during a BLAST analysis, 65 were associated with a plasmid replicon, which may facilitate their dissemination globally (Carroll et al., 2019). *Mcr -9* cassette is usually located either on a IncHI_2_ and/or IncHI_2A_ replicon or chromosomally encoded (**Table S5**) (Carroll et al., 2019; Li et al., 2020; Lin et al., 2020; Tyson et al., 2020). It is widely distributed within Enterobacteriaceae, particularly within *Enterobacter* sp (Chavda et al., 2019; Yuan et al., 2019; Kananizadeh et al., 2020; Lin et al., 2020; Ding et al., 2021)., *Citrobacter telavivum* (Ribeiro et al., 2021), *Salmonella* sp (Carroll et al., 2019; Cha et al., 2020; Tyson et al., 2020)., and *E. coli* (Kieffer et al., 2019; Khanawapee et al., 2021) (**Table S4**). It has further been reported worldwide in nine countries with Sweden and Thailand having the most reports (**Table S3**).

A database search using *mcr-9* as a template identified a chromosomal-encoded *mcr-9*-like gene within a *Buttiauxella* species isolate, a member of the Enterobacteriaceae (Kieffer et al., 2019). The identified *mcr*-like protein, MCR-BG, was isolated from *B*. *gaviniae*, and was found to have an 84% amino acid identity to MCR-9 enzyme.CR-BG, similar to *mcr-9*, has the *wbuC* gene located downstream (Kieffer et al., 2019).

Similar results were reported by Yuan et al. with a PEtN transferase isolated from *B*. *brennerae* (Yuan et al., 2019). It had 86.83% protein sequence identity to MCR-9 at a 100% coverage (Yuan et al., 2019). Further, the *wbuC* located adjacent the *mcr-9* gene was found to be homologous to that of *Buttiauxella* with an 86% amino acid identity at a 98% coverage (Yuan et al., 2019). This evidence suggests that *mcr-9* may have originated from *Buttiauxella* species and disseminated to the rest of Enterobacteriac*eae* family.

The dissemination of *mcr-9* may have been aided by its association with IS elements flanking the cassette (Lin et al., 2020). The *mcr-9* cassette has been found flanked with IS*903* and IS*903*-like elements upstream as well as IS*1R*, IS*26*-like, and IS*15DI*I located downstream in multiple reports (Kieffer et al., 2019; Yuan et al., 2019; Lin et al., 2020; Ding et al., 2021). The first report of the *mcr-9* cassette encoded the *wbuC* gene, a TCS encoding *qseC* and *qseB* genes, ΔIS*1R* and a remnant of Δ*silR*, encoding a transcriptional regulatory protein, all of *mcr* ch are loc*mcr* d downstream of the *mcr-9* gene. The *mcr-9* cassette is thereafter flanked by intact IS*903* elements (Yuan et al., 2019). Though two copies of an intact IS element should be able to form a composite transposon or a circular intermediate, an inverse-PCR assay failed to detect any intermediates (Yuan et al., 2019). However, the identification of IS*903*-like elements associated with *mcr-9* genes suggest the acquisition of the cassette through an IS*903* dependent mechanism. In some reports, the 3’region of *mcr-9* is adjacent to *copS* (three copper resistance membrane-spanning proteins that includes *rcnA*, *rcnR* and a domain-containing DUF4942) (Li et al., 2020; Ding et al., 2021).

The *mcr-10* gene was first identified in an *Enterobacter roggenkampii* clinical strain in China (Wang C et al., 2020) and has only been reported there since. It has a nucleotide sequence identity and amino acid sequence identity of 79.69% and 82.93% respectively to *mcr-9* (Tyson et al., 2020). Therefore, like *mcr-9*, the *mcr-10* gene shares a significant amino acid identity to the chromosomally encoded *mcr*-like PEtN transferase of *Buttiauxella* species (Wang C et al., 2020).

The *Mcr-10* gene has been identified on IncFIA and IncFIB plasmids (Wang C et al., 2020; Sato et al., 2021; Xu et al., 2021; Yang et al., 2021) and adjacent to a *xerC* gene, which encodes a XerC type tyrosine recombinase found to mobilize adjacent genetic components such as antimicrobial resistance genes (Wang C et al., 2020). Thus, the *mcr-10* gene would be presumed to have disseminated across Enterobacteriaceae family but has only been identified commonly in *Enterobacter roggenkampii* (Lei et al., 2020; Wang C et al., 2020; Xu et al., 2021) and once in *Cronobacter sakazakii* (**Table S4**) (Yang et al., 2021). Wang et al. and Xu et al. suggest that *E*. *roggenkampii* is an important reservoir of the *mcr-10* gene (Wang C et al., 2020; Xu et al., 2021).

In the initial identification of *mcr-10*, the gene was adjacent XerC and flanked by two intact IS*903* elements which could form a composite transposon with the potential to mediate mobilization of the *mcr-10* gene (Wang C et al., 2020). The analysis of the immediate genetic environment of the IS*903* element found the absence of direct repeats that are generated during an insertion event; thus, the acquisition of the region bracketed by the IS*903* elements was not due to an insertion event (Wang C et al., 2020). The presence of an a ΔIS*Ec36* downstream the *mcr-10* gene was reported to make it impossible to identify the recombination sites that the XerC-type tyrosine recombinase recognizes (Wang C et al., 2020). Thus, the acquisition events of *mcr-10* into p*MCR-10*_09006 remains unknown (Wang C et al., 2020). *Mcr-10* has thereafter been (Lei et al., 2020) identified with various insertion sequences, including complete and truncated remnants of IS*26* and IS*Kpn26*, complete sequences of IS*Ec36* and IS*1*, a ΔIS*Ecl1*-like elements and recently, a new IS element designated IS*Crsa1* (Lei et al., 2020; Yang et al., 2021). The identification of various IS elements downstream *mcr-10*-*xerC* suggests that this region might be a hotspot for insertions of MGEs (Wang C et al., 2020; Xu et al., 2021). The acquisition of *mcr-10* is presumed to be through site-specific recombination (Yang et al., 2021), however, evidence of mobilization of *mcr-10* is scarce.

## Dissemination of mcr genes

**Figure 5:**
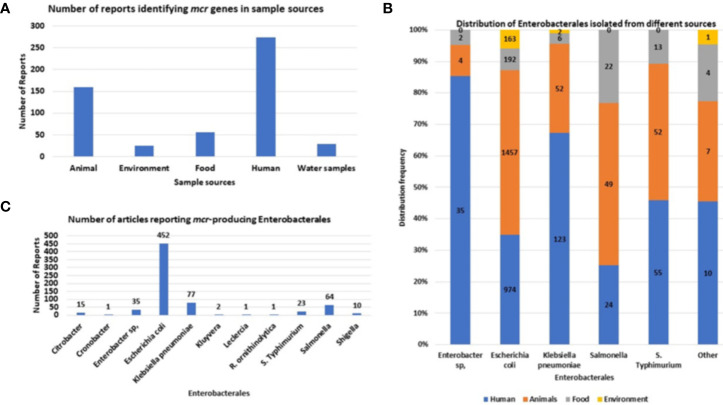
The sources and types of Enterobacteriaceae species identified to be harboring mcr genes. **(A)** Number of articles reporting samples harboring mcr-positive Enterobacteriaceae isolates. **(B)** Distribution of MCR-producing Enterobacteriaceae species per sample source. **(C)** The number of articles reporting each mcr-positive Enterobacteriaceae species.

*Mcr* genes are well disseminated within Enterobacteriaceae, being frequently identified within *E. coli*, *K. pneumoniae* and *Salmonella* species, in that order. Other Enterobacteriaceae species that have been identified with *mcr* genes include *Cronobacter* species, *Citrobacter* species, *Enterobacter* spp.*, Kluyvera* species, *Leclercia* species, *Raoultella ornithinolytica*, and *Shigella* species (**Figure 5**).

Initially, *mcr* genes were commonly identified in animals. However, after evaluating the number of reports collected from 2016 to 2021, the most common report of *mcr*-positive Enterobacteriaceae isolates is from human specimens (**Figure 5A**). This is due to the increased use of colistin in the clinical setting and the elimination of colistin use in poultry, shifting the focus away from poultry samples to the dissemination of *mcr* genes in the clinical setting. Colistin was banned for poultry use in India, China, South Africa, European Union, Thailand, Brazil and many more (Apostolakos and Piccirillo, 2018; Shen et al., 2020), however countries such as Nigeria and Bangladesh, the use of colistin is not regulated (Ahmed et al., 2020; Anyanwu et al., 2021; Anyanwu et al., 2022). *E. coli* is the most common *mcr*-positive isolate (**Figure 5C**) and is usually isolated from animal and human feces samples.

In human specimens, *mcr* genes are frequently isolated from *E*. *coli*, *K. pneumoniae*, *and S. *Typhimurium from mostly stool and urine samples (**Table S2**. In animals, the most common samples that were screened for *mcr* genes, include feces and meat, which commonly harbored *E. coli, K. pneumoniae* and *Salmonella* isolates (**Table S2**). Other sources identified includes the environment, which are mostly made up of wastewater samples which includes water samples from sewer sheds and wastewater plants treatments facilities. Other environmental samples includes soils and aquaculture such as duck and fish. In the environment, *mcr*-positive isolates comprised only three species: *E. coli*, *K. pneumoniae*, and *Kluyvera* sp (**Table S2**). Lastly, *mcr*-positive species have been also isolated from livestock meat and vegetable samples, being isolated from mostly packaged meat and fresh vegetables in retail stores. These species include: *Cronobacter* sp., *Enterobacter* sp., *E. coli*, *K. pneumoniae*, *Salmonella*, and *S.* Typhimurium (**Figure 5B**).

Amongst the 70 countries identified with *mcr* genes, countries such as Thailand harbored six *mcr-*genes (*mcr-1, mcr-3.mcr-6, mcr-7, mcr-8* and *mcr-9*) and China harbored eight genes (*mcr-1, mcr-3, mcr-4, mcr-5, mcr-7, mcr-8, mcr-9* and *mcr-10).* Other countries such as the USA, Turkey, Spain, Nigeria, Korea, Japan, Italy, France, England, Czech Republic, Cambodia, Brazil, Belgium, and Bangladesh harbored three to four *mcr-*genes, each inclusive of the *mcr-1* variants (**Figure 3B**, **Table S3**). Most *mcr* genes have been reported in studies from China (in **Figure 3C**), which is due to the large volumes of articles being published on *mcr* epidemiology from China.

It has thereafter been seen in China, that clones within *mcr-*positive *E. coli*, which includes ST744, ST410, ST10, ST43, ST101 and ST206, have been identified in all four sources: food, environment, animals, and humans (**Table S4**). These clones have, however, also disseminated globally, where ST744 has been identified in humans in eleven countries, in food in five countries, in animals in five countries and in the environment only in China. The *mcr*-positive *E. coli* ST10 strain is the most widely distributed within *E. coli*. It has been identified in animals in seventeen countries, humans in sixteen countries, the environment in five countries, and in food in five countries. *Mcr-*positive *E. coli* clones within each country are usually found in both animals and humans, seen with ST744, ST69, ST117, ST131 and ST354 in Italy.

*S.* Typhimurium is also well distributed globally, with the *S.* Typhimurium ST34 strain identified in China (Yi et al., 2017), Colombia (Saavedra et al., 2017), Denmark (Litrup et al., 2017), Germany (Borowiak et al., 2019), and the United Kingdom (Doumith et al., 2016), in both animals and humans. In China, the *S.* Typhimurium ST34 strain has been identified in both humans and animals, and in food samples and human specimens in Germany. Similar results are seen in the monophasic variants of *S.* Typhimurium serovars (S.) 1,4,[5],12:i:-, and S. 4,[5],12:i:-, where ST34 was the only clone identified with *mcr* genes (Litrup et al., 2017; Migura-Garcia et al., 2020). The S. 1,4,[5],12:i:- ST34 strain has only been identified in Portugal in animals, food and humans, and the S. 4,[5],12:i:- has been identified in Belgium, Canada, Italy, Switzerland and the United States in animals and humans (**Table S4**).

The direct transmission of *mcr-*positive Enterobacteriaceae (MCRPE) isolates from animals and humans is discussed in this review (See *Risk factors*) and the data mentioned above, highlights colistin-treated livestock as the reservoir of *mcr*-genes and the route of transmission to humans.

**Figure 6:**
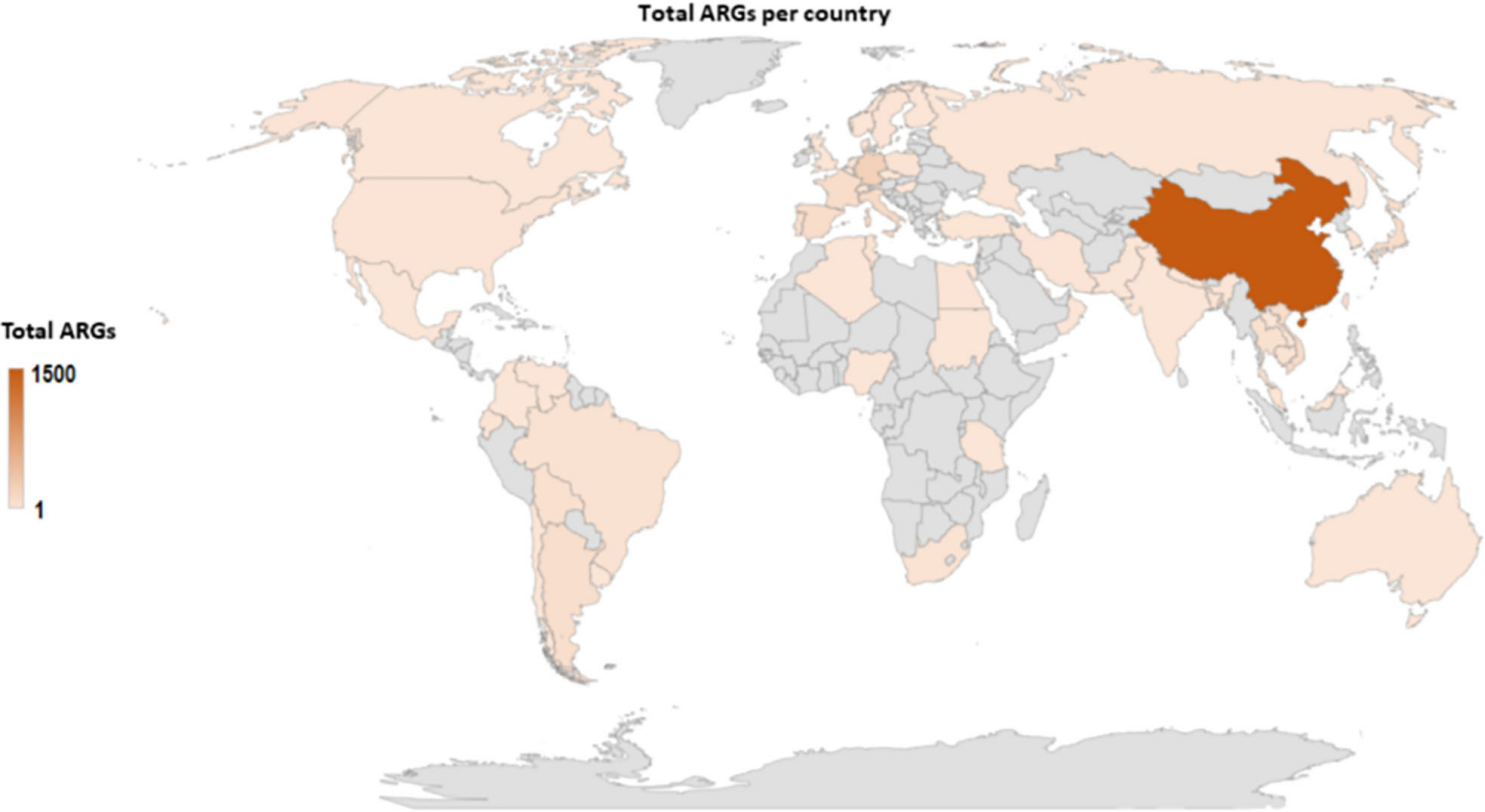
Global map showing the geographical distribution of other antibiotic resistance genes found in identified MCRPE isolates.

*Mcr-*positive isolates are commonly identified harboring other resistance genes such as *bla* genes, *mdfA, aadA12, sul123*, etc. Thus, similar to *mcr* genes (**Figure 3C**), other antimicrobial resistance genes (ARGs) are predominantly located in China (**Figure 6**), followed by Germany, Denmark, and England. The data however is biased towards China, because the large volumes of *mcr* epidemiology papers published from China. Notably, several important ARGs are co-hosted by MCRPE isolates (**Figure 3A**). A Table showing the distribution of *mcr*-positive isolates hosting other ARGs is shown in **Table S5**.

## MCR -protein structures and mechanisms of action

MCR-1 is a 541 amino acid, integral membrane protein made up of two domains: a C-terminal periplasmic catalytic domain and an N-terminal 5’-helix transmembrane domain (Liu et al., 2018; Wei P et al., 2018). The transmembrane (TM) domain is made up of 5-membrane spanning α-helixes with an overall positive charge because of positively charged residues, which interact with the negatively charged phospholipid head groups of the membrane bilayer (Wei P et al., 2018). The TM domain anchors the protein into the inner membrane and is connected to the catalytic domain through a bridging helix (Wei P et al., 2018)’. Sun et al. found that the acquisition of the transmembrane domain allows for the correct localization of PEtN transferases within the periplasm and that the deletion of this domain, impairs MCR-2’s ability to confer colistin resistance (Sun et al., 2017b). The catalytic domain is made up of both positively and negatively charged residues, where the negatively charged residues create the binding pockets and allow them to be buried within the domain (Wei P et al., 2018).

**Figure 7:**
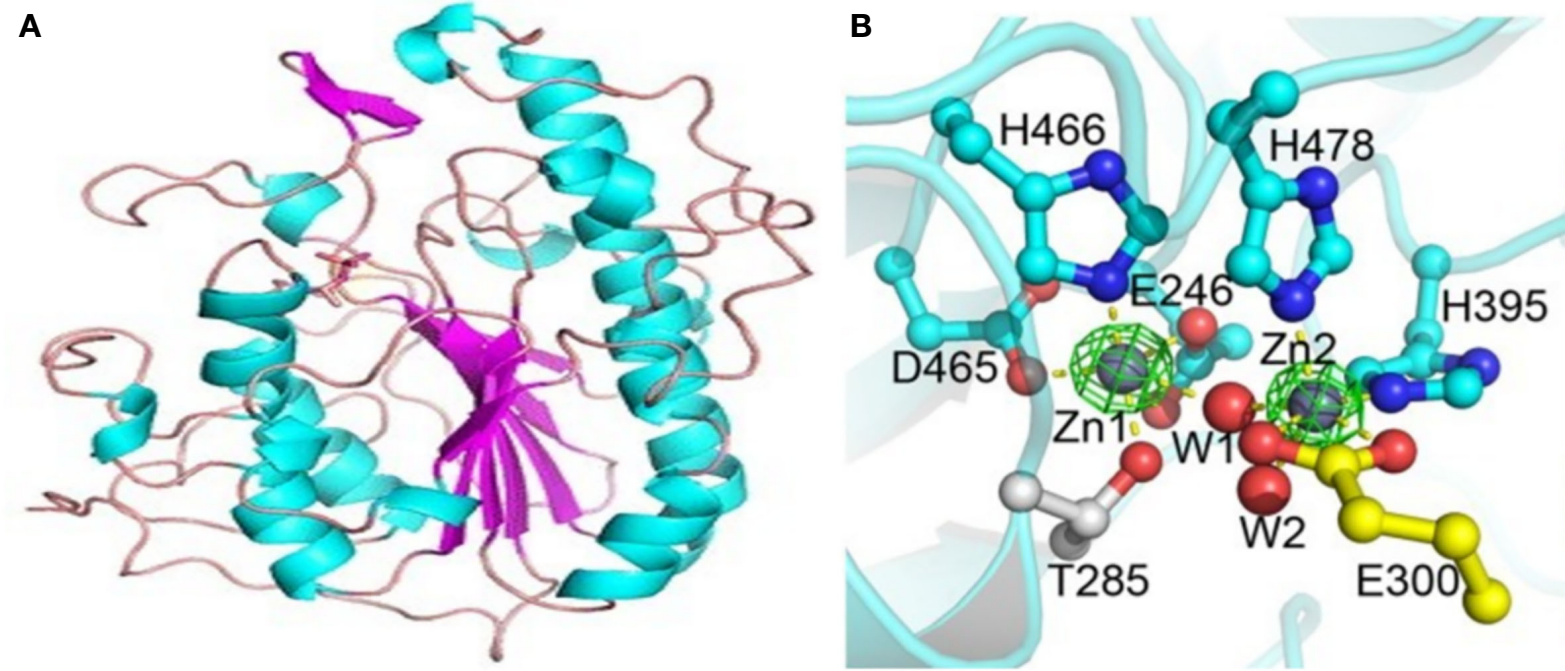
Structure of the catalytic domain of the MCR-1 protein and the five important residues required for its catalytic activity. **(A)** The MCR-1’s hemispherical shape composed of the β-α-β fold made up of helices (cyan), strands (purple) and loops (salmon). Image was obtained from Stojanoski et al. (2016). **(B)** A ball and stick model showing the conserved active site residues of MCR-1 consisting of Asp **(D)**465, Glu **(E)**246, His **(H)**466, His**(H)**395 and Thr **(T)**285, coordinated to the zinc ions (Zn1 and Zn2) and water molecules (W1 and W2). Image was obtained from Ma et al. (2016).

The overall shape of MCR-1 is a hemispherical shape composed of several β-α-β-α motifs made up of β-strands sandwiched between α-helical structures (Hu et al., 2016; Stojanoski et al., 2016). The catalytic domain has an alkaline phosphatase family α/β/α fold, also with a hemispheric shape equipped with a zinc binding pocket containing a conserved phosphothreonine-285 residue (**Figure 7**) structure (Hu et al., 2016; Stojanoski et al., 2016). The pocket is common to PEtN transferases and alkaline phosphatase; each enzyme, however, differs in the orientation and number of zinc molecules (Stojanoski et al., 2016). The pocket is proposed to be critical for the nucleophilic attack of the phosphate of the donor PE substrate by stabilizing the alkoxide of the Thr285 (Stojanoski et al., 2016). The Thr285 residue, is the catalytic nucleophile that acts as both a nucleophile and a PEtN acceptor during the catalytic mechanisms. The residue is critical to MCR-1’s function as mutations to this residue significantly decrease MCR-1’s activity (Stojanoski et al., 2016; Hinchliffe et al., 2017). The catalytic domain of MCR-1 is further made up of six cysteine residues that form three disulphide bonds, Cys281/Cys291, Cys356/Cys364 and Cys414/Cys422 (Stojanoski et al., 2016) that stabilize and anchor the β-α-β-α motifs (Hu et al., 2016). These disulphide bonds are conserved, and equivalents are present in both LptA and EptC transferases (Stojanoski et al., 2016), although LptA has four more cysteine residues.

The zinc binding pocket is in the active site of MCR-1 and is made up of conserved residues viz., Asp465, Glu246, His466, His395, and Thr285, and is made up of zinc molecules (**Figure 7**) (Gao et al., 2016; Stojanoski et al., 2016). The conserved residues are critical for the substrate binding of MCR-1 (Gao et al., 2016) and mutations in these amino acids abolish the MCR-1 activity, lowering the colistin MIC value down to control levels (Hu et al., 2016). The five conserved residues are well conserved in MCR-1, LptA, EptC and other alkaline phosphatase family members, although the nucleophile residue may differ between enzymes (Hinchliffe et al., 2017). MCR-1 has three zinc molecules clustered around the active site: Zn1 is buried within the active site and coordinated by the phosphate oxygen of Thr285; it is conserved in LptA and EptC (Stojanoski et al., 2016). Zn2 is coordinated by His395, bound to a phosphate oxygen of Thr285 and three water molecules, forming a trigonal bipyramidal configuration, (**Figure 7**) (Stojanoski et al., 2016). Hu et al. found that Zn2 is not critical for maintaining MCR-1 activity (Hu et al., 2016).

This may be because it is located on the surface of the active site. A structural comparison between LptA, EptC and MCR-1 found that the Zn2 site is less conserved. Hence, Hinchliffe et al. concluded that an intact Zn2 site was not a prerequisite for MCR-1’s catalytic activity (Hinchliffe et al., 2017). However, His395 residue is important for the structure and activity of MCR-1. The Zn3 molecule, unlike the other zinc molecules, is not coordinated by the conserved residues but is tetrahedrally coordinated by four water molecules. The seven water molecules found in the active site, coordinating Zn2 and Zn3, are embedded in the protein through hydrogen bonding (Stojanoski et al., 2016). The catalytic domain is thus a zinc-rich area (**Figure 7B**), suggesting that MCR-1 may be able to attract zinc ions at different levels into the domain (Hu et al., 2016). The role of zinc in mediating lipid A modification, the catalytic mechanism, was evaluated by measuring the colistin MIC in the presence and absence of EDTA. Hinchliffe et al. found clear reductions in colistin MIC values after EDTA treatment and concluded that the presence of zinc in the active site was important for MCR-1 function (Hinchliffe et al., 2017).

MCR-1 mediates the transfer of PEtN from the primary phosphatidylethanolamine (PE) to lipid A moiety at the 4’-phosphate group through a ‘ping-pong’ mechanism seen across MCR family regardless of their evolutionary placement (Hu et al., 2016; Gao et al., 2016; Xu et al., 2018; Zhang et al., 2019; Zhang et al., 2019a). Wei et al. and Liu et al. investigated the molecular mechanism involved in this process (Wei P et al., 2018; Liu et al., 2018). Wei et al. identified that the active site within the catalytic domain is made up of two binding pockets: the PEtN binding pocket and the Lipid A binding pocket (Wei P et al., 2018). These binding pockets are in close proximity and are integrated within each other (Wei P et al., 2018). Wei et al. observed that ethanolamine was a good analogue of PEtN and D-glucose for lipid A (Wei P et al., 2018). Ethanolamine (ETA) is a good analogue as it mimics the covalent PEtN-enzyme intermediate. Lipid A is made up of two glucosamine units that are attached to acyl chains (Wei P et al., 2018). Thus, monosaccharides and disaccharides can mimic a Lipid A molecule as they are both hexacyclic compounds (Wei P et al., 2018; Liu et al., 2018).

The PEtN binding pocket that accommodates ETA is made up of Glu246, Thr285, Asn329, Lys333, His395, Asp465, His466 and His478 residues (Wei P et al., 2018). This pocket is responsible for the binding of phosphatidyl-ethanolamine (PE), which is stabilized through hydrogen bonding with Asn329, a water molecule, and phosphorylated Thr285 (Wei P et al., 2018). Analysis of the entry of ETA into the pocket showed that the pocket undergoes conformational changes to adjust to the substrate. These included a 50° rotation of the His395, accomplished by breaking a hydrogen bond with a water molecule (wat7) and creating a new hydrogen bond with a new water molecule (wat8) (Wei P et al., 2018). This releases the wat7 molecule from the PEtN binding pocket and thus, collectively, the conformational changes create room to accommodate the substrate, ETA. The entry of ETA further deprotonates the phosphorylated Thr285 residue, forming a PEtN molecule and a nucleophilic state at Thr285 (Wei P et al., 2018). The Zn1 molecule subsequently facilitates the stabilization of the nucleophilic state of Thr285 that allows that residue to thereafter attack the PEtN, creating a PEtN enzyme intermediate. This constitutes the first step of the MCR-1-mediated catalytic process (Wei P et al., 2018).

Wei et al. and Liu et al. investigated the mechanisms behind PEtN-transfer reaction with a lipid A analogue, D-glucose, identified within the Lipid A binding pocket (Wei P et al., 2018; Liu et al., 2018). This pocket is made up of the following residues: Thr283, Ser284, Tyr287, Pro481 and Asn482, that is located near the Thr285 residue. The analysis of D-glucose found that D-glucose was held in the pocket by Thr285, Ser284 and Asn482 and was flanked by Tyr281 and Pro481 forming a sandwich structure of a π-π conjugative interaction (Wei P et al., 2018; Liu et al., 2018). The importance of the residues in both PEtN and Lipid A binding pockets in MCR-1 activity were evaluated through mutation construction. Mutations within Thr285, Asn329, Lys333, Glu246, His398, Asp465, His466 and His478 of the PEtN binding pocket and Tyr281 and Pro481 of the Lipid A binding pocket, resulted in a decrease in colistin MIC values (Wei P et al., 2018). However, these mutations did not disrupt the protein expression and membrane localization of the MCR-1 protein. The mutations of Tyr281 and Pro481 in the lipid A binding pocket highlights the importance of this pocket in the MCR-1 activity. Wei et al. suggests that it could bind to the glucosamine group of Lipid A and allows for the transfer of the reactive phosphate group from PEtN enzyme intermediate to lipid A (Wei P et al., 2018). Thus, the second step of the catalytic process therefore involves the transfer of PEtN to Lipid A that is situated in a nearby pocket (Wei P et al., 2018).

The MCR-1 catalytic domain is therefore made up of a PEtN binding pocket and a Lipid A binding pocket that mediate the transfer of PEtN from the PEtN binding pocket to lipid A moiety at the 4’-phosphate group (Hu et al., 2016; Zhang et al., 2017). This activity is validated through MALDI-TOF mass spectrometry (MS), provides *in-vivo* evidence of the MCR-1 activity (Gao et al., 2016; Anandan et al., 2017). This is accomplished by evaluating the LPS lipid A MS profile in the presence and absence of *mcr-1* expression as adding PEtN increases Lipid A mass units (+123) (Gao et al., 2016; Xu et al., 2018; Dortet et al., 2018). In the absence of *mcr-1* expression, there is a single Lipid A peak (m/z = 1797.4) and in the presence of *mcr-1* expression, there are two unique peaks. The first peak is the wildtype bis-phosphorylated, hexa-acylated lipid A (m/z = 1797.4) and the second peak is the PPEtN-4’ (m/z = 1920.5) (Xu et al., 2018).

MCR-1 has thus proven to have PEtN transferase activity and has a sequence and structural similarity to EptC and LptA PEtN transferases in *Campylobacter jejuni* (Fage et al., 2014) and *Neisseria meningitidis* (Wanty et al., 2013), respectively (Stojanoski et al., 2016; Gao et al., 2016; Hinchliffe et al., 2017). The active sites of the three PEtN transferases are highly conserved, i.e., the phosphorylated catalytic nucleophile Thr285, disulphide bonds, zinc binding pockets and the conserved active site residues (Hinchliffe et al., 2017). Therefore, these three sites are important for MCR-1 function; these conserved mechanistic activities and binding pockets will allow for the use of MCR-1 inhibitors to also inhibit chromosomally encoded PEtN transferases, restoring colistin susceptibility in acquired and intrinsically resistant bacteria (Stojanoski et al., 2016).

All ten MCR proteins (MCR-1 to MCR-10) encode a putative PEtN transferase made up of two domains- transmembrane helix and a periplasmic domain with the catalytic center (Yin et al., 2017; Borowiak et al., 2017; Wang et al., 2018b; Yang et al., 2018; Zhang et al., 2019a; Zhang et al., 2019b; Wang X et al., 2019; Carroll et al., 2019; Xu et al., 2021). These proteins further have the same conversed properties required for PEtN activity, i.e., the active residues (E246, T285, K333, H395, D465, H466, E468 and H478) and the six cysteine residues required for the three-sulphide bonds (Xavier et al., 2016; Sun et al., 2017b; Teo et al., 2018; Wang et al., 2018b; Wang et al., 2018c). A phylogenetic analysis of all MCR proteins shows that MCR-1, MCR-2, MCR-6 and *Neisseria* LptA were part of a parallel evolutionary event for functional acquisition of colistin resistance during some environmental selection pressure, i.e., the intensive use of colistin in animals feed (Sun et al., 2017b; Wang et al., 2017; Wang Y et al., 2020; Islam et al., 2020). Thereafter, MCR-3, MCR-4, MCR-7, and MCR-9 have similar amino acid sequences (Kieffer et al., 2019; He et al., 2021), and further, are closely related at a structural level (Carroll et al., 2019). However, MCR-5, MCR-8 and MCR-10 have a low identity to the other *MCR* proteins, though a sequence alignment shows that all ten MCR proteins each encode the conserved active residues, six cysteine residues and each is capable of LPS modifications (Borowiak et al., 2017; Wang et al., 2018b; Wang X et al., 2019; Carroll et al., 2019). Zhang et al. further showed that MCR-5 and MCR-1/2 have different interdomain relationships, and their domains (TM1-MCR-5, TM5-MCR-1, TM2-MCR-5 and TM5-MCR-2) are mostly incompatible (Zhang et al., 2019b). MCR-5, however, has 100% amino acid sequence identities to PEtN transferases identified in *Pigmentiphaga* genus and *Cupriavidus gilardii*. The first 404 amino acids of the MCR-5 protein also have a high identity to a hypothetical protein from *Pseudomonas aeruginosa* (Borowiak et al., 2017).

All MCR proteins therefore, like MCR-1 mediate the transfer of PEtN from the PEtN binding pocket to the Lipid A moiety at the 4’-phosphate group. This reduces the MCR-producing microorganism’s susceptibility to colistin (Kieffer et al., 2018; Long et al., 2019). Thus, the difference in nucleotide and amino acid sequences between the MCR proteins have no impact on their PEtN activity. However, Yang et al., found that *mcr* genes, i.e., the *mcr-*variants, may encode compensatory mutations that mitigate the expression of *mcr* genes or might affect PEtN’s activity (Yang et al., 2020). This was found with *mcr-4.3* identified by Teo et al. which encodes two missense mutations, V179G and V236F, that inactivated the PEtN activity (Chavda et al., 2018; Teo et al., 2018). A comparative alignment of the MCR-4.3 to MCR-1 and MCR-2 found that the active residues were conserved in MCR-4.3 (Teo et al., 2018). However, an MS spectrum of lipid A in *mcr-4.3*-expressing isolates showed *mcr-4.3* failed to modify lipid A (Chavda et al., 2018). The transformation of *E. coli* with an *mcr-4.3* expressing vector resulted in no difference in colistin MIC values, suggesting that *mcr-4.3* does not confer colistin resistance (Chavda et al., 2018; Teo et al., 2018). Thus, some compensatory mutations in *mcr* seen in *mcr-4.3* significantly altered the PEtN transferase activity of *mcr-4.3* (Chavda et al., 2018).

## Fitness cost and compensatory evolution

The acquisition of *mcr-1* bearing plasmids has been shown to have a beneficial effect on the host, improving bacterial survival in the presence of colistin treatment (Nang et al., 2018). The acquisition and expression of the *mcr-1* gene results in the incorporation of MCR-1 into the bacterial membrane and the phosphoethanolamine (PEtN) modification of the lipopolysaccharides (LPS) (Li et al., 2017). However, multiple studies have shown that the expression of *mcr-1* imposes a fitness cost by placing an energy burden on the host (He et al., 2017), impairing cell growth, and diminishing bacterial fitness (Li et al., 2017). Andersson et al. explained that a significant fitness cost is seen when the susceptible strain outcompetes the resistant strain in an antibiotic free environment (Andersson, 2006). The imposed fitness cost of a conjugative plasmid harboring a resistance gene has been shown to be because of various factors such as the resistance mechanisms, the bacterial species, and the antibiotic (Nang et al., 2018).

Evaluating the cost of colistin resistance on resistant strains, it is shown that certain chromosomal mutations within genes such as *mgrB* have no significant fitness cost on *K. pneumoniae* (Cannatelli et al., 2015) whilst genes such as *pmrB*, do (Giordano et al., 2019). Giordano et al. found that the expression of the *mcr-1* gene imposes less of a burden on *K. pneumoniae* than the mutated *pmrB* gene (Giordano et al., 2019). The chromosomal mutations within *pmrAB*, *acrab*, *phoPQ*, and *mgrB* genes have been shown to mediate colistin resistance through the phosphorylation of lipid A (Cheng et al., 2016; Phan et al., 2017; Cannatelli et al., 2017). The a*crab* genes encode a multidrug efflux system that confers resistance to a wide variety of agents (Mmatli et al., 2020). Tietgen et al. discovered that the fitness costs imposed by *mcr-1* plasmid carriage, such as growth rates and cytotoxicity, could be species-specific (Tietgen et al., 2018). Particularly, the acquisition of an *mcr-1*-harboring plasmid in *K. pneumoniae* has been shown to impose a significant fitness cost on the host (Nang et al., 2018), but multiple studies have shown that the acquisition of an IncI_2_ plasmid, in various sizes, carrying *mcr-1* had no significant cost on the host (Zheng et al., 2017; Wang R et al., 2018). This may be plasmid and/or species specific or may be due to acquired compensatory mutations within the IncI_2_ plasmid. However, the overexpression of *mcr-1* was seen to result in profound changes in the architecture of the outer membrane, resulting in the loss of membrane structural integrity and causing leakage of cellular cytoplasm, resulting in cell death (Li et al., 2017; Yang Q et al., 2017).

Yang et al. found that the overexpression of *mcr-1* imposes a significant cost by decreasing the growth rate, causing significant membrane degradation and moderate fitness loss’ (Yang Q et al., 2017). They came to this conclusion through evaluating the effects of *mcr-1* expression on the relative fitness of *E. coli* TOP10 and found that with increasing levels of *mcr-1* expression resulted in a significant fitness burden on the host (Li et al., 2017; Yang Q et al., 2017). An analysis of the cellular morphology in the *mcr-1* overexpressed strains using a transmission electron microscopy showed cell architecture alterations and a complete loss of cellular morphology. The overexpression of *mcr-1* altered the structural integrity of the outer membrane and further impaired the cell membrane (Li et al., 2017). They finally found that the embedding of MCR-1 into the outer membrane and the PEtN modifications of Lipid A were the leading factors contributing to fitness cost and membrane degradation. Therefore, the expression of *mcr-1* in host strains is tightly controlled to regulate the *mcr-1* fitness cost (Yang Q et al., 2017).

This was further seen in *mcr-3* expression, where expression was shown to significantly impair the cell wall integrity and decrease the electron density of *mcr-3* producing *E. coli* 94. Yang et al. evaluated the impact of *mcr-3* expression on bacterial fitness using two *mcr-3* variants, *mcr-3*.1 and *mcr-3*.5 (T488I, M23V, A456E) (Yang et al., 2020). The study showed that although the expression of both variants imposed a fitness cost and impaired the cell wall integrity, the expression of *mcr-3*.5 was less costly (Yang et al., 2020). Yang et al. showed that the amino acid substitutions A457V and T448I, seen in *mcr-3*.5, had strong compensatory effects when introduced in *mcr-3*.1 (Yang et al., 2020). The introduction of these substitutions, individually, resulted in an increased fitness of up to 45%. However, double substitutions demonstrated a negative epistasis. Yang et al. further evaluated the fitness cost imposed by *mcr-3* expression and compared it to that of *mcr-1* and found that the expression of both *mcr* genes had an impact on the bacterial fitness; however, the *mcr-3*.1 and *mcr-3*.5 variants imposed less of a fitness cost (Yang et al., 2020).Zhang et al. compared the physiological effect of *mcr-1, mcr-3*, and *mcr-4* expression in recombinant strain of *E. coli* MG1655 and observed inhibited bacterial growth with each *mcr* gene, with *mcr-4* having less of a physiological impact (Zhang et al., 2019a).

Expression of *mcr* gene is therefore quite toxic for the bacterial host, and the acquisition of an *mcr-*bearing plasmid thereafter results in compensatory adaptation that allows for the maintenance of high-cost conjugative plasmids (Dahlberg and Chao, 2003; Yang YQ et al., 2017; Ma et al., 2018). Dahlberg and Chao found that the cost of plasmid carriage is reduced over long-term culture because of compensatory mutations (Dahlberg and Chao, 2003). The host chromosome or plasmid evolves compensatory mutations that, in the case of plasmids, enhance the fitness of the host, and in the case of chromosomal mutations, aid the host in evolving towards new growth conditions by decreasing the plasmid carriage cost (Dahlberg and Chao, 2003). This has been seen in multiple studies where *mcr-1* bearing plasmids initially imposed a biological cost on the transformant. However, overtime, the cost of plasmid carriage in long-term cultures was largely compensated for and plasmids were stably maintained through passages (Zhang et al., 2017; Li et al., 2017; Tietgen et al., 2018; Ma et al., 2018; Giordano et al., 2019).

Ma et al. investigated the potential mechanisms involved in compensatory adaptation through comparative genomics and identified single nucleotide polymorphisms in several genes (Ma et al., 2018). Amongst the genes identified in this study are *dnaK*, which encodes a molecular chaperon involved in chromosomal DNA replication, and *cpoB*, which encodes an RHS repeat protein involved in maintaining the cell envelope integrity during cellular division (Ma et al., 2018). These two genes are located on the chromosome and have non-synonymous single nucleotide polymorphisms (SNPs). The role of these genes in reducing plasmid carriage cost is unknown and may represent novel mechanisms (Ma et al., 2018). The study further shows that bacteria may use different strategies to reduce the fitness cost of plasmid carriage under different environmental conditions (Ma et al., 2018).

In the absence of antibiotics (colistin) selective pressure, *mcr-1* bearing plasmids have been shown to be less maintained and that the complete elimination of the *mcr-1* gene within a population is possible (Nang et al., 2018). Nang et al. performed a plasmid stability assay and found that in the absence of colistin, there was a gradual loss of the *mcr-1* plasmid, there was a decrease in its maintenance within the population. This may be due to the instability of *mcr-1* harboring plasmids (Nang et al., 2018). Arcilla et al. have shown the complete elimination of *mcr-1* bearing bacteria in travelers returning to their home country after a month (Arcilla et al., 2016).

## Risk factors for acquiring mcr genes

### Use of colistin in livestock

Antimicrobials are commonly administered by farmers to food animals such as pigs, calves, and poultry for prophylaxis and therapeutics purposes, and as a growth promoter (Carrique-Mas et al., 2015; Nguyen et al., 2016; Monte et al., 2017). Studies around the world have shown that the use of colistin as a growth promoter results in a high frequency of *mcr-1* positive, colistin-resistant *Enterobacteriaceae* because of the excessive use of colistin in the farming environment. The selection pressure led to the acquisition and spread of *mcr-1* genes (Liu et al., 2016; Nguyen et al., 2016; Monte et al., 2017; Trung et al., 2017; Shen et al., 2018; Hille et al., 2018; Clemente et al., 2019). The presence of *mcr-1* in food animals had a serious potential to spread MCRPE to humans *via* foodborne and direct contact transmission routes (Liu et al., 2016; Trung et al., 2017; Shen et al., 2018).

The pharmaceutical form of colistin is indicated for oral administration and is present in a powder or solution form (Hille et al., 2018). Studies have shown that the drug is poorly absorbed in the digestive tract after oral administration and can be excreted in high levels through the feces (Rhouma et al., 2015; Peng et al., 2021). This results in the presence of colistin or its active metabolites in the environment alongside the MCRPE isolates from the animal feces (Shen et al., 2018). Rhouma et al. found that the movement of colistin through the gastric passage of pigs, produced degradation products of colistin made of several metabolites that have lost some colistin side chains. These metabolites however had increased antimicrobial activity as compared to the original form of colistin (Rhouma et al., 2015). Xia et al. (2019) measured the concentration of colistin in animal feeds and fresh manure in five swine farms and found that the average concentration of colistin in animal feed on farm 4 was 60 mg/kg, which was scientifically higher than other farms. The fresh manure samples collected on farm 4 had the highest concentration of 17,383 µg/kg. Other farms had colistin concentrations in their fresh manures ranging from 140 µg/kg in farms 1, 2 and 5, to 7,529 µg/kg in farm 3 (Xia et al., 2019). The researchers also discovered a strong relationship between colistin concentration and the relative abundance of *mcr-1* genes in fresh manure (Xia et al., 2019). The use of this manure as a fertilizer in agriculture or feeding farmed fish can result in the contamination of agriculture and, in some cases, the aquatic environment, polluting rivers and lakes (Luo et al., 2017; Shen et al., 2018).

This therefore led to the ban on colistin use for growth promotion and disease prevention in the food industry to preserve their effectiveness in human medicine as their use contributes to the rising threat of antibiotic resistance (World Health Organization, 2017). This colistin ban however, was only adopted in some countries. Randall et al. (2018) also demonstrated that discontinuing colistin in farming can result in the elimination of *mcr-1*. This longitudinal study evaluated the presence of *mcr-1* in pig feces and slurry at two time points (Randall et al., 2018). At the beginning of the study, the majority of the pigs were colonized by *mcr-1* producing *E. coli*. Twenty months after cessation of colistin use and implementation of hygiene and other measures, *mcr-1* producing *E. coli* was not detected in samples. These results thus highlight that the use of colistin is the driving force behind the spread of colistin resistance genes and its stability in the population (Randall et al., 2018). *Mcr-1* has however, been identified on conjugative plasmids encoding other resistance genes such as *floR* and *sul123*. This allows for the use of other antibiotics such as chloramphenicol and sulfonamide to simultaneously co-select the *mcr-1* gene, allowing for its stability and dissemination within the conjugative plasmid (Nguyen et al., 2016; Haenni et al., 2016; Skov and Monnet, 2016). This, thus, creates severe challenges for controlling the selection and subsequent transmission of *mcr-1* genes (Yang et al., 2016).

### Dissemination of *mcr-*contaminated feces into the environment

Luo et al. cultured a *mcr-1* producing *Raoultella ornithinolytica*, a member of the *Enterobacteriaceae* family commonly found in soil, aquatic, and botanical environments, in retail vegetables in China. They emphasized that using animal excrements contaminated with colistin residuals and/or MCRPE can cause contamination and spread of MCRPE to fresh vegetables (Luo et al., 2017).. Further, in fish-livestock integrated farms, fish are fed animal manure, which acts as a fertilizer for aquaculture ponds (Dang et al., 2011). The deposit of manure/animal excrements with colistin residuals into fish ponds or the environment favors the selection and growth of colistin-resistant bacteria (Shen et al., 2019). In Lebanon, there is evidence of the aquatic environment being affected by anthropogenic waste including sewage and agricultural waste (Hmede and Kassem, 2018; Sulaiman and Kassem, 2019; Hassan et al., 2020; Dandachi et al., 2020; Kassem, 2020). There is, however, little global epidemiological data on the contamination of rivers and lakes by MCRPE-contaminated animal excrements and thus, no evidence of the spread of MCRPE to the aquaculture (Cabello et al., 2017; Shen et al., 2018). However, the presence of *mcr-1*-producing *E. coli* has been reported in duck and fish samples in China. Shen et al. (2019) found a potential spread of *mcr-1* producing *E. coli* from aquaculture supply chain to humans through seafood (Shen et al., 2018; Shen et al., 2019; Hassan et al., 2020). Other examples of the spread of MCRPE into the environment includes the identification and isolation of MCRPE in blowflies (*Chrysomya* sp.) (Yang QE et al., 2019) and black kites (Tarabai et al., 2019). Both cases are of public concern because of the potential transmission into human communities.

Yang et al. (Yang QE et al., 2019) showed that blowflies may serve as an environmental reservoir and vector of MCRPE between animals, humans, the environment, and waste (landfills and sewage water) (Yang QE et al., 2019). The presence of MCRPE in Black kites in Russia highlights the spread of *mcr-1* genes into both wildlife and the environment. Tarabai et al. (2019) hypothesized that the acquisition of MCRPE was either through contact with the Biysk Municipal landfill or through their food, which was commonly found along the Biya River near their nest. Black kites can thereafter spread MCRPE along their long migratory pathways (Tarabai et al., 2019). The *mcr-1* gene is commonly found on transferable plasmids, and thus the presence of *mcr-1* producing isolates in the microbiota of insects and wildlife increases the possibility of horizontal transfer of *mcr-1* genes alongside other resistance genes within the microbiota. This increases the environmental gene pool and the spread of multi-drug resistant (MDR) pathogens within insects and wildlife (Yang QE et al., 2019).

### Dissemination of *mcr* genes to humans

There are therefore multiple reservoirs highlighted above with the potential of spreading towards humans because of the close association between humans and the environment. A study in Vietnam showed that the farmers that are in contact with animals exposed to colistin have a high risk of being colonized by MCRPE (Trung et al., 2017). Trung et al. (2017) found that chickens associated with colistin administration had a high carriage of *mcr-1* producing isolates and the colonization of humans was through exposure to the chickens. The chicken faecal samples had a high prevalence of *mcr-1* (59.4%) and zoonotic transmission prevalence was 34.7% in farmers. Another possible route of transmission of MCRPE from animals exposed to colistin, was foodborne transmission (Liu et al., 2016; Malhotra-Kumar et al., 2016; Agnoletti et al., 2018). Monte et al. found that chicken meat was acting as a reservoir of *mcr-1* producing *E. coli*. A study performed by Shen et al. (2018) found a positive correlation between *mcr-1* producing *E. coli* carriage in the human normal flora and the consumption of colistin-exposed farm animals, which included pork and sheep. The correlation analysis also proposed a possible transmission of *mcr-1* producing *E. coli* from aquatic food to humans (Shen et al., 2018; Hassan et al., 2020). This data is a single example that highlights the presence of MCRPE in food-producing animals and the transmission route of MCRPE from colistin-exposed animals to humans, which impacts public health care.

### Risk-factors

After the first detection of *mcr-1* by Liu et al. (2016), *mcr-1* genes have been detected in animals, the environment, and humans. Initially, the detection of *mcr-1* genes in humans was associated with the risk factors mentioned above: contact with farm animals, ingestion of colistin-exposed farm animals, or fresh vegetables. Yang et al. identified *mcr-*1 producing *E. coli* and *K. pneumoniae* in market retail fruits, a significant report because fruits are consumed without cooking and processing unlike meat and vegetables. This is potentially a high-risk source of *mcr-1* (Yang F et al., 2019). Epidemiology studies that identified *mcr-1* genes in the clinical settings were accompanied with a questionnaire to identify the MCRPE source. In countries such as Finland, where the use of colistin is restricted for rare clinical indications, the first detection of the *mcr-1* gene was identified in a healthy male with previous history of travelling abroad, 6 months before fecal sampling (Gröndahl-Yli-Hannuksela et al., 2018). Traveling to other countries has been shown to increase the risk of acquiring *mcr-1* positive isolates, particularly in patients who consumed meat while traveling abroad (Gröndahl-Yli-Hannuksela et al., 2018; Henig et al., 2019). The use of antibiotics prior to hospitalization or infection has been seen as an important risk factor for a multidrug resistant organism infection (Henig et al., 2019). An epidemiological and clinical study performed by Wang et al. (2017) reported that *mcr-1* producing *E. coli* can acquire other resistance genes, and thus previous use of antibiotics such as carbapenems and fluroquinolones was associated with an increased risk of infection by *mcr-1* producing *E. coli* (Wang et al., 2017). Thus, the administration of antibiotics in clinics simultaneously promotes the co-selection and the preservation of colistin resistance genes (Lu et al., 2019).

An interesting possible transmission of *mcr-1* producing *E. coli* is between companion animals and humans. A worker at a pet shop with no prior antibiotic use or travelling abroad was identified carrying an *mcr-1* producing *E. coli* (Zhang et al., 2016). A fecal screening of the pets residing where the man worked identified 4 dogs and 1 cat with an *mcr-1* positive isolate. Zhang et al. (2016) suggested companion animals as a possible reservoir of colistin-resistant *E. coli*, facilitating the spread of colistin resistance genes within the community.

## Current and future perspectives on treating mcr-mediated colistin resistant infections

### Clustered regularly interspaced short palindromic repeats (CRISPR)

Clustered regularly interspaced short palindromic repeats (CRISPR) has been exploited and developed within molecular biology as a site-specific tool for genetic engineering (Hryhorowicz et al., 2017), and in regards to resistance, as a tool to deliver a programmable DNA nuclease (CRISPR-Cas9) into an MDR-pathogen to eliminate the resistance gene or plasmid (Dong et al., 2019). Dong et al. (2019) developed a conjugative CRISPR-Cas9 system that aimed to remove plasmids harboring the *mcr-1* gene from bacteria. The system targeted the *mcr-1* gene and the authors found that the formation of DSBs during the elimination of the resistance gene resulted in the elimination of the whole plasmid in recipient cells (Dong et al., 2019). This thereafter sensitized the recipient cells to colistin. Dong et al. (2019) further found that the cells are thereafter immune against the *mcr-1* gene. Wang et al. (Wang P et al., 2019) used the same concept but developed the tool to remove both *mcr-1*-harboring plasmids and MDR plasmids present in recipient cells using partial sequences of the targeted plasmids. He et al. (2021) used the tool to eliminate both chromosomal and plasmid-borne *mcr-1* genes in *E. coli.* The CRISPR-Cas9 system was designed to target IS*Apl1*, which eliminated *mcr-1* harboring plasmids, and in the case of chromosomally encoded *mcr-1*, cell death was seen. Similar results were achieved (Dong et al., 2019), where recipient cells further acquired immunity against the acquisition of the exogenous *mcr-1* containing plasmid (He et al., 2021). These studies show that CRISPR-Cas9 system is an efficient tool for plasmid curing and to sensitize clinical isolates to antibiotics *in vitro* (Dong et al., 2019; Wang P et al., 2019). This tool can be optimized for therapeutic application for the elimination of antibiotic genes from resistance reservoirs such as animal guts with prior exposure to antibiotics, the human microbial flora or the bacteria in natural settings such as wastewater, farms or hospital settings (Dong et al., 2019).

The CRISPR-Cas9 system is however made up of highly anionic nucleic acids and proteins that cannot penetrate the cell membrane into cells and thus requires a delivery vehicle. Sun et al. (Sun L et al., 2017) explored Cathelicidins, which are short cationic antimicrobial peptides, as a plasmid delivery system. The study evaluated the use of BMAP-27 to increase the efficiency of plasmid transfer of the CRISPR-Cas9 system and found that pCas::*mcr* exhibited better, efficient, and specific antimicrobial effects with the help of BMAP-27 (Sun L et al., 2017). The CRISPR-Cas9 system is a powerful tool for genome editing, its development into targeting multiple genes with one use (Wang P et al., 2019), could aid in the removal of *mcr* genes in resistance reservoirs and in clinical settings to restore colistin activity.

In the meantime, there are multiple studies evaluating existing antibiotics for activity against MCR-producing isolates and synergy combinations, summarized in **Table 1**.

**Table 1:** Summary of current and future perspectives on treating *mcr*-mediated colistin resistance.

Treatments	Description		
CRISPR-Cas			
*Novel antibiotics:*			
Eravacycline	Broad-spectrum synthetic tetracycline with bactericidal activity		
Plazomicin	Broad-spectrum, semi-synthetic aminoglycoside		
Artilysin ^®^Art-175	Engineered destabilizing peptide derived from endolysins which degrade cell walls.		
*Antimicrobial peptides*			
Bacteriocins	Ribosome-synthesized antimicrobial cationic peptides that creates pores on cytoplasmic membrane		
AA139 and SET-MM3	AMPs with concentration dependent bactericidal activity against pathogens, irrespective of their antibiotic susceptibility profiles.		
Human cationic AMPs: LL-37, α-defensin (HD5), β-defensin (HDB5)	Widely distributed proteins within the human immune system. They have the same mechanisms of action as colistin and unaffected by MCR proteins		
Humanized monoclonal antibodies	Derivates of human immune system found not to be affected by MCR proteins		
*Peptide nucleic acid and Phosphorodiamidate morpholino oligomers (PPMOs)*			
Peptide nucleic acid	Antisense molecules designed to target mcr *-1* mRNA preventing translation of MCR -1 proteins. This restores colistin sensitivity		
Phosphorodiamidate morpholino oligomers (PPMOs)			
*Natural compounds*			
Osthole coumarin	MCR-1 inhibitor with a bactericidal activity in combination with colistin		
Honokial	Exhibits a bactericidal activity when combined with colistin against colistin resistant organisms irrespective of their mechanisms		
Isoalantolactone			
Calycosin			
Eugenol			
*Effux pump inhibitors*			
CCCP		EPI able to restore colistin sensitivity respective of molecular resistant mechanisms	
*FDA-approved drugs*			
Pentamidine		Associates with the outer membrane by inhibiting OS core antibiotic adjuvant biosynthesis	
Zidovudine		Nucleoside reverse transcriptase inhibitor with antibacterial when in combination with colistin activity against MDR Enterobacteriaceae strains	
Azidothymidine			
Sulphonamide compounds		Synergistic activity with colistin independent of colistin-resistance mechanism	
Polymyxin derivative NAB739		Antibiotic adjuvant with bactericidal activity against MDR pathogens when used in combination with meropenem, retapamulin and rifampicin,	
Compound PFK-185		Anti-tumor drug with bactericidal activity when used with colistin	
*Combination therapy*: restores colistin bactericidal activity			
Rifampicin		Azithromycin + Rifampicin	Clarithromycin
Rifabutin		Tigecycline	minocycline
Aztreonam + Amikacin		Amikacin	

### Antimicrobials agents effective against *mcr-1* producing isolates

There are multiple novel antibiotics that have been found to have activity against *mcr-1*-producing isolates, including eravacycline (Fyfe et al., 2016; Zhao et al., 2019) and plazomicin (Denervaud-Tendon et al., 2017). Eravacycline is a broad-spectrum synthetic tetracycline that has been proven to be effective against clinically important MDR pathogens, including both Gram-negative and Gram-positive bacteria (Zhao et al., 2019). Fyfe et al. (2016) found that eravacycline had a bactericidal effect against MCR-producing isolates from *Enterobacteriaceae*. The study further showed that although the overexpression of *mcr-1* increased colistin MIC values by 64-folds, there was no effect on eravacycline susceptibility (Fyfe et al., 2016). Eravacycline activity was thereafter evaluated against 336 isolates collected from hospitals across China and was found to exhibit a good efficacy against all strains (Zhao et al., 2019). These isolates included Extended spectrum β-lactamase (ESβL) and carbapenemase-producing *Enterobacteriaceae*, MCR-producing *Enterobacteriaceae* and *A. baumannii*, vancomycin-resistant *Enterobacteriaceae*, β-lactamase producing *Haemophilus influenzae*, penicillin-resistant *Streptococcus pneumoniae* and lastly, methicillin-resistant *Staphylococcus aureus* (MRSA), thus showing a positive potential to treat current drug-resistant bacterial infections (Zhao et al., 2019). This, however, also puts eravacycline at risk of being overused.

Plazomicin is a novel broad-spectrum semi-synthetic aminoglycoside that was derived from Sisomicin. It has been shown to have activity against a broad spectrum of MDR pathogens, including ESβL- and carbapenemase-producing isolates and fluroquinolone-resistant isolates (Denervaud-Tendon et al., 2017). Denervaud-Tendon et al. (2017) evaluated the bactericidal activity of plazomicin against colistin-resistant Enterobacterial strains, which harbored different colistin resistance mechanisms i.e., chromosomal-encoded, *mcr-1*-expressing, and intrinsically resistant strains. Plazomicin activity was further compared with other aminoglycoside antibiotics viz., amikacin, gentamicin, and tobramycin. Plazomicin displayed potent activity against the clinical colistin-resistant Enterobacterial strains irrespective of their resistance mechanisms. However, those that were intrinsically resistant (*Serratia, Proteus, Morganella* and *Hafnia)* had a higher MIC value (Denervaud-Tendon et al., 2017).

Denervaud-Tendon et al. also found that amongst all aminoglycoside antibiotics tested, plazomicin was the most potent (Denervaud-Tendon et al., 2017). They lastly also found that plazomicin’s activity is restricted by aminoglycoside resistance mechanisms such as the 16S rRNA methylase-encoding gene, which resulted in an elevated plazomicin MIC value (>128 mg/L) (Denervaud-Tendon et al., 2017). Plazomicin, however, like other aminoglycosides, has a rapid bactericidal activity with favorable chemical and pharmacokinetics properties and thus could be incorporated into therapeutic treatments against MCR-producing bacterial infections (Denervaud-Tendon et al., 2017).

Artilysin ^®^ Art-175 is a novel engineered enzyme-based experimental therapeutic derived from endolysins produced by lytic bacteriophages. These endolysins degrade the bacterial cell wall resulting in cell lysis, which occurs at the end of the bacteriophage infection cycle (Gerstmans et al., 2016). Artilysin ^®^ Art-175 is made up of the outer membrane destabilizing peptide, which is fused to an endolysin, it targets the anionic lipopolysaccharide molecules of the outer membrane of Gram-negatives. Schirmeier et al. (2018) evaluated the potential of Art-175 against pan-drug resistant isolates, including colistin-resistant *mcr-1* producing *E. coli* and further determined the potential of cross-resistance between colistin and Art-175. Art-175 was highly bactericidal against the tested isolates, which were more susceptible to Art-175 than the colistin-susceptible isolates (Schirmeier et al., 2018). Hence, there is no cross-resistance between the two peptides, highlighting Art-175 as a potent solution against MCR-producing isolates (Schirmeier et al., 2018).

### Antimicrobial peptides

Bacteriocins are ribosome-synthesized antimicrobial peptides (AMPs) produced by both Gram-positive and Gram-negative bacteria. Studies have shown that bacteriocins produced by lactic acid bacteria (LAB) are cationic peptides with bactericidal activity that act on the cytoplasmic membrane of susceptible microorganisms (Drider et al., 2016). The LAB bacteriocins create pores in the membrane, resulting in intracellular damage. Al Atya et al. (2016) evaluated the combinations of nisin and enterocin DD14 LAB bacteriocins with colistin to eradicate colistin-resistant *E. coli* strains in either planktonic states or in biofilm. Whilst colistin and the bacteriocins were ineffective as monotherapy against both planktons and biofilms, colistin-nisin and colistin-enterocin DD14 combination were able to reduce the number of colony-forming units (CFU) of strains in both planktonic and biofilm states. The triple combination of all three molecules, colistin-nisin-enterocin DD14, completely eradicated all *E. coli* strains, including colistin-resistant phenotype (Al Atya et al., 2016). LAB bacteriocins alone cannot penetrate the outer membrane. However, with the disruption of the LPS *via* colistin activity, the LAB bacteriocins may thereafter act upon the cytoplasmic membrane (Al Atya et al., 2016). Al Atya et al. (2016) therefore highlights LAB bacteriocins as a potential novel adjunctive treatment for colistin-resistant *mcr-1* producing *E. coli* infections.

There have been multiple reports of AMPs that have been developed for treating MDR pathogens, which have a similar mode of action as colistin (van der Weide et al., 2019). Van der Weider et al. evaluated the antimicrobial activity of two novel AMPs, AA139 and SET-MM3. AA139 originates from a marine lugworm, *Arenicola marina*, AMP arenicin-3. SET-MM3 is a synthetic tetra-branched peptide linked by a lysine core (van der Weide et al., 2019). The antimicrobial activity of these AMPs was evaluated against a collection of clinically and genotypically diverse *K. pneumoniae* with different antimicrobial susceptibility profiles. Both AMPs, AA139 and SET-M33, further showed a concentration-dependent bactericidal effect across all isolates, meaning that the susceptibility profile of both AMPs was unaffected by the resistance profile and colistin susceptibility of the isolates (van der Weide et al., 2019). The AMPs were effective against the colistin-resistant strains, and no cross-resistance was observed between the AMPs and colistin. Thus the antimicrobial activity of AA139 and SET-M33 should further be investigated across other Gram-negatives to evaluate its spectrum of bactericidal activity (van der Weide et al., 2019).

Cross-resistance between several human cationic AMPs (CAMPs) and polymyxins has been previously reported in PEtN transferase-producing *Haemophilus ducreyi and Campylobacter jeyuni* but rarely in *Enterobacteriaceae*. These CAMPs are part of the intrinsic human immune system and act against Gram-negative bacteria by disrupting the outer and inner membrane through electrostatic interaction, similar to colistin’s mechanism of action (Dobias et al., 2017). Dobias et al. (2017) selected a few widely distributed CAMPs in humans, LL-37, α-defensin 5 (HD5) and β-defensin 3 (HDB3) and evaluated whether cross-resistance between CAMPs and polymyxin would be observed in *mcr-1* producing *E. coli.* Susceptibility of *E. coli* to the different CAMPs selected varied depending on the CAMP molecule rather than the entire CAMP family. It further showed that the production of MCR proteins did not confer cross-resistance with the human CAMPs selected (Dobias et al., 2017).

However, the CAMPs activity studied by Dobias et al. (2017), compared to other studies (Napier et al., 2013), shows that factors such as bacterial species, level of resistance, and colistin resistance mechanisms may affect the bactericidal levels of CAMPs (Napier et al., 2013; Dobias et al., 2017). Therefore, although *mcr* production did not affect the bactericidal activity of CAMPs against *E. coli*, it needs to be evaluated across *Enterobacteriaceae* and other Gram-negative bacteria to see how different colistin resistance mechanisms affect the CAMPs’ bactericidal effect. Guachalla et al. (2018) further evaluated the effectiveness of other derivates of the human immune system against a clinical *mcr-1* producing *E. coli* strain. This includes humanized monoclonal antibodies that have been previously shown to target the LPS O25b antigen that is associated with *E. coli* ST131-H30 (Guachalla et al., 2018). The expression of *mcr-1* does not affect the binding of ASn-4 to the LPS’s O-antigen and the integration of the complement membrane-attack complex (MAC) into the LPS-modified outer membrane (Guachalla et al., 2018). This means the expression of *mcr-1* in *E. coli* ST131-H30 does not affect the functionality of the human immune system, nor does it not aid the strain *via* immune evasion. The authors thereafter suggested the use of antibodies targeting the LPS’s O-antigen as an alternative strategy to combatting MDR infections against *mcr-1* positive isolates (Guachalla et al., 2018).

Other novel compounds produced to manage MCR-producing isolates are antisense agents with antimicrobial properties; this include phosphorodiamidate morpholino oligomers (PPMOs) and peptide nucleic acids (PNAs) (Nezhadi et al., 2019). These antisense molecules are designed to target mRNA, prevent translation, and restore antimicrobial sensitivity. Daly et al. (2017) designed peptide-conjugated PPMOs targeted to *mcr-1* mRNA (MCR-1 PPMO) of clinical *mcr-1*-positive *E. coli* strains and showed that these molecules were able to make the strains re-sensitive to polymyxins by MCR-1 inhibition (Daly et al., 2017). Similar results were achieved by Nezhadi et al. (2019) with PNAs, designed for inhibition of *mcr-1* translation. They *(* Nezhadi et al., 2019 *)* showed that the introduction of PNA resulted in a 95% reduction of *mcr-1* expression, measured by RT-PCR, highlighting the efficacy of PNAs (Nezhadi et al., 2019). Daly et al. (2017) evaluated the antisense approach in a sepsis model using mice and found that a combined therapy of MCR-1 PPMO with colistin reduced morbidity and bacterial burden in the spleen at 24hr. Thus, antisense agents may be effective therapeutics, either alone by targeting an essential gene resulting in cell death (Daly et al., 2017), or in combination with colistin (Daly et al., 2017; Nezhadi et al., 2019) to treat *mcr-1*-producing isolates. However, these molecules have varying toxicity effects and more *in-vivo* research is required prior to clinical application (Nezhadi et al., 2019).

### Natural compounds

Other novel compounds that have been reported to have synergistic effects with colistin against *mcr-1* producing isolates includes the novel MCR-1 inhibitor, Osthole (7-methoxy-8-(3-methyl-2-buteryl) coumarin. Osthole (OST) is a natural compound derived from the dried root and rhizome of *Cnidium monnieri* (Zhou et al., 2019). Zhou et al. (2019) evaluated the effects of OST on the inhibition of MCR-1 enzyme and the mechanisms behind the inhibition. The colistin-OST combination had a synergistic effect as a mouse-thigh-infection model showed that the combination exhibits a bactericidal activity against *mcr-1*-producing *Enterobacteriaceae*, significantly reducing the bacterial load in the thighs following subcutaneous administration (Zhou et al., 2019). Using a molecular dynamic stimulation, Zhao et al. (Zhou et al., 2019) found that OST could localize in the binding pocket (residue 330-350) of MCR-1, blocking the substrate and reducing MCR-1’s biological activity. OST therefore inhibits MCR-1 in *Enterobacteriaceae*, making colistin potent (Zhou et al., 2019). Due to the conservation of MCR proteins, OST may have the same inhibition efficiency across the other MCR genes.

Natural/organic compounds identified with a synergistic effect on colistin include Honokiol (Guo et al., 2020), Isoalantolactone (IAL) (Lu et al., 2020), Calycosin (Liu et al., 2020) and Eugenol (Wang et al., 2018). Honokiol was derived from the dry skin and bark of *Magnolia officinalis (* Guo et al., 2020 *)*, IAL is the main sesquiterpene lactone from Radix inulae and other plants (Lu et al., 2020). Calycosin is a flavonoid from a direct root extract of the traditional Chinese medicinal herb, Radix astragali (Liu et al., 2020). Lastly, Eugenol is a phenylpropanoid, an essential oil isolated from plants (Dos Santos et al., 2021). Each of these compounds, when combined with colistin, decreased colistin MICs and increased colistin’s bactericidal activity against colistin-resistant isolates. Eugenol decreased the expression of *mcr-1* (Wang et al., 2018) and honokiol bound directly to the active site of MCR-1, inhibiting its activity (Guo et al., 2020). Guo et al. (2020) and Liu et al. (2020) showed that the natural compounds, honokiol and calycosin, in combinations with colistin, respectively reduced the load of bacteria and improved viability in animal models.

### Efflux pump inhibitors

Efflux pumps play a role in reducing colistin susceptibility in *Enterobacteriaceae* (Mmatli et al., 2020). Baron et al. (Baron and Rolain, 2018) found that an efflux pump inhibitor, carbonyl cyanide 3-chlorophenylhydrazone (CCCP), was a good alternative to reverse colistin resistance in colistin-resistant isolates irrespective of their molecular resistance mechanisms. The study shows that CCCP was able to restore colistin activity across a diverse set of *Enterobacteriaceae* isolates carrying different colistin resistance mechanisms, intrinsic and acquired (*mcr-1*, *pmrAB*, *mgrB*, etc) (Osei Sekyere and Amoako, 2017; Baron and Rolain, 2018). In *mcr-1* producing isolates, it was seen that colistin-CCCP combination inhibited the transcription of the *mcr-1* gene. The mechanism behind this observation, however, is unknown (Baron and Rolain, 2018).

### FDA-approved drugs

The food and drug administration (FDA) has a library of FDA-approved drugs that can be screened for bactericidal activity on MCR-producing *Enterobacteriaceae* isolates (Prestwick Chemical, Illkirch-Graffenstuden, France). Multiple compounds such as pentamidine, zidovudine (Okdah et al., 2018), sulphonamide compounds (Okdah et al., 2018), a polymyxin derivative, NAB739 (Zhang Y et al., 2019), and compound PFK-185 (Zhang Y et al., 2019), were identified.

Zidovudine is a nucleoside reverse transcriptase inhibitor. In the 1980s, it was used as an anticancer drug (Chow et al., 2009). In 1986, its antibacterial effect was revealed (Elwell et al., 1987) and in 1987, it was used as the first antiretroviral for treatment against HIV infections (Chow et al., 2009; Peyclit et al., 2018). Peyclit et al. (2018) investigated the antibacterial activity of Zidovudine against a large number of characterized MDR *Enterobacteriaceae* strains isolated from different geographical areas, including carbapenem- and colistin-resistant isolates; Zidovudine was effective against *Enterobacteriaceae*. Despite their antimicrobial susceptibility profile, the MIC values ranged between 0.05-1.67 µg/mL (Peyclit et al., 2018).

Another antiretroviral drug that is used to treat HIV/AIDS but also has bactericidal activity against Gram-negative bacteria is Azidothymidine (AZT). Hu et al. (2019) shows that AZT, in combination with colistin, was able to eradicate MDR *Enterobacteriaceae* strains with different antimicrobial susceptibility profiles and enhance the activity of colistin. The combined therapy was also effective therapeutically in a murine peritoneal infection against NDM-1-producing *K. pneumoniae* and *mcr-1* producing *E. coli* (Hu et al., 2019).

Further, Pentamidine is an antiprotozoal drug that Stokes et al. (2017) identified as an effective antibiotic adjuvant producing synergistic combination activity against a wide range of Gram-negative bacteria. The adjuvant was identified during a library screening of non-lethal, outer membrane-active compounds (Stokes et al., 2017). The study shows that Pentamidine directly associates with the outer membrane by inhibiting the oligosaccharide (OS) core biosynthesis, releasing LPS from the outer membrane (Stokes et al., 2017). Stokes et al. (2017) further showed that Pentamidine has synergistic activity with hydrophobic antibiotics such as rifampicin, novobiocin, and erythromycin but not with hydrophilic and low-molecular weight antibiotics (Stokes et al., 2017). Although the study does not evaluate synergistic combinations with Pentamidine, Stokes et al. (2017) concluded that pentamidine can be used as an antibiotic adjuvant for treating colistin-resistant infections.

Barker et al. (2017) screened a diverse cohort of adjuvants to identify a compound that could both sensitize colistin-resistant and hypersensitize colistin-susceptible bacteria to colistin, to potentially lower the effective dosage of colistin, reducing its toxicity. The study identified three compounds that were potent modulators of colistin resistance and were able to significantly reduce MIC values in both plasmid- and chromosomal-mediated resistance mechanisms, and further hypersensitize colistin-susceptible isolates (Barker et al., 2017)

Another compound identified in the FDA-approved library with synergistic activity with colistin is sulfadiazine (SDI), a sulphonamide compound. Okdah et al. (2018) evaluated the potential of different sulphonamide compounds with potential synergistic and bactericidal activity in combination with colistin; SDI had the highest synergistic effect. The combination of SDI-colistin was effective, independent of colistin resistance mechanism, across the broad range of MDR bacterium strains tested (Okdah et al., 2018).

Zhang et al. (Zhang Y et al., 2019) identified an antitumor drug, PFK-185 and its analogs, PFK-015 and 3PO, during a screening of the clinical compound library. These compounds can exert synergize with colistin against colistin-resistant *Enterobacteriaceae* despite *mcr* expression and the antimicrobial susceptibility profile (Zhang Y et al., 2019). Zhang et al. (Zhang Y et al., 2019) found that PFK-185 had no effect on cellular morphology when used alone. However, when in combination with colistin, it enhanced the bacterium-killing effect of colistin, increasing the survival rate to 60%. The combined colistin-PFK-158 therapy had the most significant bactericidal activity, with the most significant reductions in the bacterial burdens post-*in vivo* experiments and during time-kills studies (Zhang Y et al., 2019).

The last compound reported from the FDA-approved library is a novel polymyxin derivative, NAB739, which carries only three of the five amino groups of colistin, each placed in a strategic position (Tyrrell et al., 2019). Tyrrell et al. (2019) showed that NAB739 sensitizes polymyxin-resistant strains to rifampicin and a combination of the two was synergistic against ten of the eleven colistin-resistant strains. Tyrrell et al. (2019) further showed that NAB739 was also synergistic with meropenem and retapamulin. As well, NAB739 in combination with other compounds has significant bactericidal activity against polymyxin-resistant isolates and compared to polymyxins, it has a better tolerability and efficacy (Tyrrell et al., 2019). Thus, NAB739 combination therapies may replace polymyxin for treating MDR pathogens (Tyrrell et al., 2019).

These studies (Stokes et al., 2017; Peyclit et al., 2018) however, showed that old antimicrobials listed within the FDA-approved library may be re-introduced against bacteria that are resistant to multiple antibacterial in current use (Peyclit et al., 2018).

### Combination therapy

There are multiple compounds, either antibiotics or adjuvants, which are synergistic with colistin, thus reducing the dosage of colistin required for treatment, minimizing its toxicity, overcoming colistin resistance, and ensuring maximal therapeutic efficacy (Barker et al., 2017; Hu et al., 2019). Colistin activity has been restored when it is combined with antibiotics such as rifampicin (Li Y et al., 2018; Yu et al., 2019), rifabutin, minocycline (Yu et al., 2019), amikacin (Zhou et al., 2017), tigecycline (Zhou et al., 2020) and clarithromycin (MacNair et al., 2018). In all these reports, the antibiotics were ineffective as monotherapy against MCRPE isolates. However, in combination with colistin, they resulted in significant bactericidal activity. Yu et al. (2019) found that colistin in combination with rifampicin, rifabutin or minocycline was able to eradicate XDR, NDM- and *mcr–* co-producing *E. coli in-vitro* and in mouse models. Other combinations that were confirmed effective against *mcr-1*-producing colistin-resistant *E. coli* using an animal model include tigecycline-colistin (Zhou et al., 2020), clarithromycin-colistin (MacNair et al., 2018) and a triple combination of colistin-rifampin-azithromycin discovered by Li et al. (Li Y et al., 2018). Another triple combination therapy that was discovered was colistin-aztreonam-amikacin, which was effective against both carbapenemase-producing and *mcr-1*-producing *Enterobacteriaceae* isolates (Bulman ZP et al., 2017).

Lastly, a combination therapy that was effective against both carbapenem- and colistin-resistant *Enterobacteriaceae* isolates was the β-lactam-β-lactamase inhibitor, imipenem-relebactam, combination (Carpenter et al., 2019). This combined therapy was potent against colistin-resistant carbapenemase-producing isolates, making it a potential agent against carbapenemase-producing *Enterobacteriaceae;* particularly, those that are colistin resistant (Carpenter et al., 2019).

## Conclusion

Herein, *mcr* genes are commonly identified in *E. coli*, *K. pneumoniae*, and *Salmonella* sp., with clones within these strains being disseminated globally within animals, food, the environment, and humans. *Mcr* genes were initially identified in animal samples, specifically livestock animals that were treated with colistin as a therapeutic, prophylaxis and metaphylaxis against Gram-negative bacterial infections or used to promote growth. *Mcr* genes have spread to the environment and humans through contaminated and untreated animal feces used as manure and through contaminated food-producing-animals’ products on markets, respectively. Contact with these colistin-treated, MCRPE-contaminated livestock or their feces by humans was identified as a possible transmission route. This study identified multiple possible routes of transmission of MCRPE to humans, these include routes from livestock, the environment and from livestock-treated meat. The use of colistin as a therapeutic against carbapenem-resistant *Enterobacteriaceae* infections has also shown to increase the prevalence of MCRPE in humans. The presence of clones such as *E. coli* ST744 or ST101 in all sources, including animals, the environment, food, and humans, highlights these transmission routes. This makes *mcr* a concerning antibiotic resistance gene of great interest and priority.

Further, *mcr* genes are commonly associated with mobile genetic elements. IS*Apl1* elements have facilitated the horizontal transfer of *mcr*-1 within cassettes of composite transposons. They have been also associated with IncX_4_ plasmids, facilitating their horizontal and vertical transfer across species, genera and families.

Although the *mcr* genes have different possible progenitors viz., *Moracella* sp., *Aeromonas* sp., and *Shewanella* sp. for *mcr-1*, *mcr-3* and *mcr-4*, respectively, the MCR proteins are very well conserved. Each of the 10 MCR proteins encodes a two-domain integral membrane protein with a C-terminal periplasmic domain and an N-terminal 5’-helix transmembrane domain, each MCR protein further encodes the five conserved residues i.e., E248, T286, H389, D458, and H359, located within the active site. PEtN transferase activity was found for each MCR protein, and each was able to mediate colistin resistance, although *mcr-9* expression requires a specific genetic environment to regulate its expression. The conservation seen within MCR proteins, allows for the use of similar therapies for the management of MCRPE isolates, specifically in the cases of identified MCR-1 inhibitors. Research on novel therapeutics is well-summarized in this review (**Table 1**), with techniques such as CRISPR-Cas9, peptide nucleic acids, and antimicrobial peptides, that can aid *manage mcr* -positive carbapenem resistant isolates. Other novel therapeutics were identified by reviving old FDA-approved drugs. Some were effective against MCR-producing isolates or were synergistic with colistin. These therapeutics, however, need to be further evaluated for their toxicity in humans. This will aid in alleviating the threat imposed *by mcr* -positive carbapenem isolates in the public health sector.

## Author contributions

MM undertook the systematic search, data collection and manuscript write-up; NM was a co-supervisor to the study and assisted with funding; JO designed and supervised the study, reviewed and edited the manuscript, as well as assisted with analysis of the data. All authors contributed to the article and approved the submitted version.

## Funding

This work was funded by the National Health Laboratory Service (NHLS) given to JO under grant number GRANT004 94809 (reference number PR2010486), and the National Research Foundation.

## Conflict of interest

The authors declare that the research was conducted in the absence of any commercial or financial relationships that could be construed as a potential conflict of interest.

## Publisher’s note

All claims expressed in this article are solely those of the authors and do not necessarily represent those of their affiliated organizations, or those of the publisher, the editors and the reviewers. Any product that may be evaluated in this article, or claim that may be made by its manufacturer, is not guaranteed or endorsed by the publisher.
